# Naringenin boosts Parkin-mediated mitophagy via estrogen receptor alpha to maintain mitochondrial quality control and heal diabetic foot ulcer

**DOI:** 10.1016/j.jpha.2025.101333

**Published:** 2025-05-12

**Authors:** Xin-Meng Zhou, Ying Yang, Dao-Jiang Yu, Teng Xie, Xi-Lu Sun, Ying-Xuan Han, Hai-Ying Tian, Qing-Qing Liao, Yu-Jie Zhao, Yih-Cherng Liou, Wei Huang, Yong Xu, Xi Kuang, Xiao-Dong Sun, Yuan-Yuan Zhang

**Affiliations:** a Department of Pharmacology, West China School of Pharmacy, West China School of Basic Medical Sciences & Forensic Medicine, West China School of Public Health and West China Fourth Hospital, Sichuan University, Chengdu, 610041, China; b Department of Plastic Surgery, The Second Affiliated Hospital of Chengdu Medical College, China National Nuclear Corporation 416 Hospital, Chengdu, 610051, China; c Medical College, Tibet University, Lhasa, 850000, China; d Department of Biological Sciences, Faculty of Science, National University of Singapore, Singapore, 117543, Singapore; e Department of Endocrinology and Metabolism, The Affiliated Hospital of Southwest Medical University, Luzhou, Sichuan, 646000, China; f Metabolic Vascular Diseases Key Laboratory of Sichuan Province, Luzhou, Sichuan, 646000, China; g Tianfu Jincheng Laboratory, Chengdu, 610093, China

**Keywords:** Naringenin, Diabetic foot ulcer, Mitochondrial quality control, E3 ubiquitin-protein ligase parkin, Mitophagy, Estrogen receptor α

## Abstract

Diabetic foot ulcer (DFU) is an increasing global burden due to the rising prevalence of diabetes, and no specific pharmacological targets or satisfactory drugs are currently available for this devastating ailment. In this study, naringenin (NAR) was found to accelerate diabetic wound healing in diabetic C57BL/6J wild-type (WT) mice by reducing oxidative stress, as assessed through histological assay. NAR also alleviated the inhibition of proliferation, inflammation, cell senescence, and apoptosis in HaCaT cells induced by high glucose (HG). Mechanistically, the beneficial effects of NAR on wound healing are dependent on the E3 ubiquitin-protein ligase parkin (Parkin/PRKN/Prkn). NAR upregulated the expression level of Parkin and promoted its mitochondrial translocation, thereby activating Parkin-mediated mitophagy and maintaining mitochondrial quality control (MQC). Moreover, the wound healing-promoting effects of NAR were significantly diminished in Parkin knockdown HaCaT cells and *Prkn* knockout (*Prkn*^*−*/−^) DFU mice. Inhibition of NAR binding to estrogen receptors (ERs) using tamoxifen (TAM) abolished the protective effects of NAR in HG-induced HaCaT cells. The luciferase reporter assay confirmed that NAR enhanced ERs binding to the estrogen response element (ERE), thereby upregulating Parkin transcription. Additionally, the cellular thermal shift assay (CETSA) revealed that NAR specifically bound to ERα. In conclusion, NAR promoted DFU wound healing by enhancing Parkin-mediated mitophagy via binding to ERα, highlighting its potential as a promising therapeutic candidate.

## Highlights

• NAR promotes DFU healing by maintaining MQC.
• NAR's beneficial effects are dependent on Parkin.
• NAR directly binds to ERα.
• NAR enhances the binding of ERα to ERE to upregulate the transcription of Parkin.

## Graphical abstract

**Figure:**
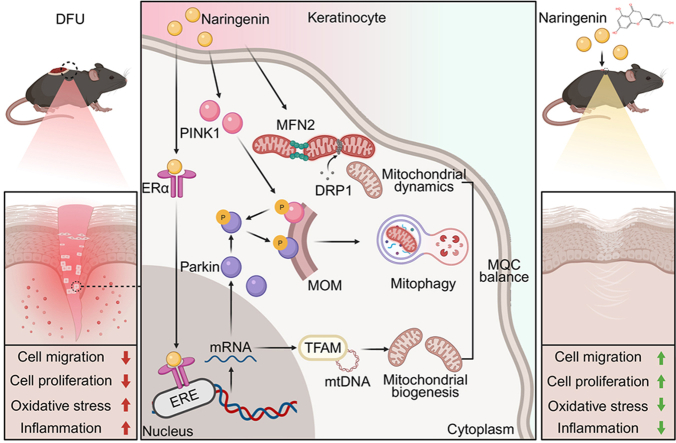

## 1 Introduction

As a common complication of diabetes, diabetic foot ulcer (DFU) is a public health problem that endangers human health [1]. As reported by a systematic review, the prevalence of DFU in the world and China was 6.3% and 4.1%, respectively [2]. DFU is the dominant reason for disability and death in diabetics, seriously influencing patients’ quality of life [3]. The five-year mortality rate is 30.5% for patients with DFU, rising to 56.6% for those who undergo above-foot amputation, compared to only9 % for breast cancer [4]. Intensive glycemic control was reported to reduce the risk of lower limb amputation by 35% in patients with type 2 diabetes [5]. However, there is no significant association between baseline hemoglobin A1c level and wound healing in patients with DFU [6]. Currently, there is a lack of specific targeted drugs for DFU. Hence, it is vital to find novel therapeutic strategies and identify potential pharmacological targets for DFU.

Mitochondrial damage was reported to be essential in nonhealing diabetic wounds [7]. Mitochondrial quality control (MQC) is an evolved sophisticated mechanism in eukaryotic cells to identify, repair, or eliminate misfolded proteins in the mitochondria or damaged mitochondria [8]. MQC is the central regulator in maintaining mitochondrial homeostasis and functions by regulating mitochondrial biogenesis, dynamics, mitophagy, etc.. MQC also alleviates mitochondrial dysfunction by suppressing oxidative stress [9]. Imbalance in MQC results in the aggravation of impaired mitochondria and is a culprit in the pathogenesis of multiple diabetic complications, such as DFU, diabetic nephropathy, and diabetic cardiomyopathy [7,10,11]. However, there are few studies on whether maintaining MQC promotes the healing of DFU. E3 ubiquitin-protein ligase parkin (Parkin/PRKN/Prkn)-mediated mitophagy can efficiently remove damaged mitochondria and alleviate oxidative stress, making it a primary mechanism of MQC [12,13]. Parkin-mediated mitophagy is indispensable for erythropoietin to alleviate renal injury in diabetic mice [14]. l-carnitine mitigates cardiac microvascular dysfunction in male db/db mice by promoting Parkin-mediated mitophagy [15]. However, the function and mechanisms of Parkin and Parkin-mediated mitophagy in DFU have not yet been thoroughly investigated.

As a member of dihydroflavonoids, naringenin (NAR) possesses multiple pharmacological activities, such as antioxidation, anti-inflammation, hepatoprotection, etc. [[16], [17], [18], [19]]. NAR alleviates myocardial damage in mice with type 1 diabetes by inhibiting the expression levels of inflammatory cytokines and upregulating the levels of antioxidant enzymes to mitigate inflammation and oxidative stress [20]. Moreover, NAR relieves the oxidative stress and retinal apoptosis in diabetic rats induced by streptomycin (STZ) [21]. However, whether NAR can reduce oxidative stress in DFU to promote diabetic wound healing, along with its underlying mechanisms, remains to be investigated. NAR exhibits strong estrogen receptor alpha (ERα) binding capacity and potent estrogen-like effects [22]. The beneficial effects of NAR inhibiting the proliferation of the non-malignant colonocytes and reducing circulating cholesterol have been demonstrated to be related to estrogen receptors (ERs) [23,24]. Gender is one of the most relevant risk factors for DFU in patients with diabetes, with a risk ratio of 2.062 for men [25]. This phenomenon may be related to the differential expression of sex hormones, estrogen-induced angiogenesis, and faster healing at wound sites in diabetic mice [26]. However, the use of estrogen is limited due to its broad pharmacological effects and cognitive reasons. There is an urgent need to find out drug candidates targeting ERs rather than estrogen. Local upregulation of ER at the wound site promotes wound healing in diabetic mice [27]. However, evidence for the direct regulation of NAR on ERα in DFU is lacking. Moreover, the effects and downstream signaling pathways of pharmacologically activating ERα in DFU remain largely unknown. The ERα signaling pathway maintains intracellular Parkin level by increasing Parkin protein stability and restoring impaired mitophagy in vascular smooth muscle cells [28]. Therefore, it was hypothesized that NAR may enhance Parkin-dependent mitophagy to maintain MQC balance via ERα, thereby promoting the wound healing of DFU.

The present study aimed to determine the effects of NAR on DFU and elucidate the molecular mechanisms. This study evaluated the effects of NAR on DFU both *in vi*vo and *in vitro*. Using C57BL/6J wild-type (WT) and *Prkn* knockout (*Prkn*^*−*/−^) diabetic mice, it was demonstrated that the absence of Parkin eliminated the positive effect of NAR on DFU wound healing, revealing that Parkin and its mediated mitophagy are essential for this process. NAR protected mitochondrial function from high glucose (HG)-induced damage by activating Parkin-mediated mitophagy and maintaining MQC balance via ERα. Direct evidence revealed that NAR could bind to ERα, enhance its binding to the estrogen response element (ERE), and upregulate Parkin transcription. These findings fill in the gap in the relationship between Parkin and its mediated mitophagy in DFU, and identify Parkin as a downstream target of ERα. This study highlighted the therapeutic potential of targeting Parkin-mediated mitophagy to restore MQC balance and promote DFU wound healing.

## 2 Materials and methods

### 2.1 Establishment of DFU mouse models using male C57BL/6J mice

Commonly used animal models for DFU include the full-thickness skin excision model [29], ischemic ulcer model [[30], [31], [32]], infected diabetic ulcer model [33,34], and neuropathic ulcer model [35]. The present study utilized the full-thickness skin excision model, which has a long history of use in DFU research [[36], [37], [38], [39]], and is frequently employed in preclinical pharmacodynamic evaluation of novel DFU therapies [40,41].

All animal experiments were conducted in accordance with the National Research Council's Guide for the Care and Use of Laboratory Animals and approved by the Medical Ethics Committee of Sichuan University, China (Approval No.: K2020047). The four-week-old male C57BL/6J mice (Beijing HFK Bioscience Co., Ltd., Beijing, China) were housed in pathogen-free conditions. Mice were fed a high-fat diet (HFD) for 90 days, followed by a daily intraperitoneal injection of 50 mg/kg STZ (Rhawn, Shanghai, China) in citrate buffer for five consecutive days. On the 14th day post-injection, blood samples were collected from the tail veins, and fasting blood glucose (FBG) level was measured using a one-touch blood glucose meter (Sinocare, Changsha, China). Notably, mice with FBG level above 200 mg/dL were confirmed as diabetic mice and used for further skin puncture. For the Sham group (*n* = 6), mice were fed a standard chow diet (CD) (Chengdu Dossy Experimental Animal Co., Ltd., Chengdu, China) for 90 days and intraperitoneally injected with citrate buffer daily for five days. Subsequently, all mice were anesthetized with isoflurane (RWD Life Science Co., Ltd., Shenzhen, China). The back hair of mice was removed with an electric clipper, followed by the application of depilatory cream. The skin was then gently cleaned with phosphate-buffered saline (PBS). Afterward, a round full-thickness wound with a 6-mm diameter was made on the dorsum of the mice by a sterile biopsy punch [29,42]. Diabetic mice were divided into four groups: control group, 1% NAR group, 5% NAR group, and 10% NAR group, with six mice in each group. NAR (PubChem CID: 439246; CAS: 67604-48-2) with a purity of above 98% was commercially obtained from Sangon Biotech Co., Ltd. (Shanghai, China). Based on the solubility of NAR in blank ointment (BO), concentrations of 1%, 5%, and 10% (*m/v*) were selected for *in vivo* experiments. NAR was incorporated into the BO, composed of polyethylene glycol (Sangon Biotech Co., Ltd.) and ultra-pure water, to prepare NAR ointments at different concentrations (1%, 5%, and 10%). BO was applied daily around the wounds of mice in the Sham and control groups, while NAR ointments were applied to the wounds of mice in the NAR groups for 10 consecutive days (from day 0 to day 9). Digital images were taken on the day of surgery and every other day post-injury for 10 days. The wound area was quantified using ImageJ (National Institutes of Health (NIH), Bethesda, MD, USA), and the wound closure rate was calculated as the percentage of the original wound area.

Following the 10-day treatment period, all mice were sacrificed, and skin tissues within 1 cm from the wound edges were collected. The fresh skin samples were divided into two equal-sized sections down the middle. One section was fixed in 4% paraformaldehyde (PFA) (Sangon Biotech Co., Ltd.) and stored at room temperature (RT). The other section was embedded into optimal cutting temperature frozen section agent (SAKURA, Nagano, Japan), frozen in liquid nitrogen, and stored at −20 °C.

### 2.2 Transgenic mice generation and treatments

To explore whether the effects of NAR on wound healing are Parkin-dependent, male four-week-old *Prkn*^*−*/−^ mice were used, which were kindly provided by Professor Jun Zhou from Nankai University (Tianjin, China). The heterozygous mice were bred in-house, and *Prkn*^*−*/−^ mice were obtained. Genotyping was carried out using the following primers: WT-forward primer (FP), CAGAAGTGATGGGTTCACACTGATG; WT-reverse primer (RP), CTTCAGGCTTTACAGGCCATCC; *Prkn*^*−*/−^-FP, GCACTTGTCATACCCTAAGAGCTTC; and *Prkn*^*−*/−^-RP, CTTCAGGCTTTACAGGCCATCC. Notably, four-week-old male *Prkn*^*−*/−^ mice were randomly divided into three groups (*n* = 5): Sham group, control group, and NAR group. Mice in the control and NAR groups were intraperitoneally administered with STZ (50 mg/kg) in citrate buffer for five consecutive days. No HFD was used to establish DFU mouse models since systemic *Prkn*^*−*/−^ mice had impaired intestinal lipid absorption and could not exhibit metabolic stress induced by HFD [43]. The Sham group received an intraperitoneal injection of citrate buffer for five consecutive days. FBG measurements, the standard for diabetes, and the methods of skin puncture were consistent with those used for C57BL/6J male mice. BO was applied daily around the wounds of mice in the Sham and control groups, while a 5% NAR ointment was applied to the wounds of mice in the NAR group for 10 days. Samples were harvested using the same method for C57BL/6J mice.

### 2.3 Histological analysis

After being fixed overnight in 4% PFA, the skin samples were dehydrated, immersed in paraffin, and then cut into 6 μm-thick slides. The hematoxylin and eosin (H&E) staining were applied to the slides to visualize the tissue morphology. All images of the stained paraffin-embedded sections were captured using a light microscope (Zeiss, Oberkochen, Germany).

### 2.4 Immunohistochemistry (IHC) staining

Briefly, serial paraffin-embedded tissue sections of skin samples were dewaxed and hydrated. Antigen retrieval was performed using citrate buffer in a pressure cooker. The slides were incubated with antibodies against caveolin-1 (Cav-1) (Sangon Biotech Co., Ltd.), proliferation marker protein Ki-67 (Abcam, Cambridge, UK), nicotinamide adenine dinucleotide phosphate (NAD(P)H): quinone oxidoreductase 1 (NQO1) (Sangon Biotech Co., Ltd.), and Parkin (Cell Signaling Technology, Danvers, MA, USA) at RT for 2 h. Afterward, they were incubated with secondary antibodies (Invitrogen, Carlsbad, CA, USA) at RT for 2 h. The samples were thereafter reacted with a 3,3′-diaminobenzidine (DAB) solution (Beijing Zsgb Bio, Beijing, China) to visualize signals, followed by counterstaining with hematoxylin. All images were captured using a light microscope (Zeiss).

### 2.5 Sulforhodamine B (SRB) assay

After being treated with NAR for 24 h, HaCaT cells were fixed at 4 °C for 1 h with pre-cooled 10% (*m*/*v*) trichloroacetic acid. Then, the cells were washed with ultra-pure water five to six times and dried at RT. After 10-min staining with 0.4% (*m*/*v*) SRB (Sigma-Aldrich, St. Louis, MO, USA) solution, 1% acetic acid was used to wash away the unbound dye solution. After being dried at RT, 10 mM Tris base was used to dissolve the dye. The optical densities at 540 nm were then measured by the SpectraMax 190 microplate reader (Molecular Devices, San Jose, CA, USA). Data were presented as the percentage of live cells compared to the 0 μM group.

### 2.6 Cell culture and treatment

SRB assay results (Fig. S1) indicated that NAR exhibited no cytotoxic effects on HaCaT cells at concentrations below 90 μM. To determine whether NAR could exert effects at lower concentrations, 0.12, 0.37, 1.1, and 3.3 μM were selected for *in vitro* experiments. HaCaT human keratinocyte cells, kindly provided by Professor Shuyu Zhang from Sichuan University (Chengdu, China), were cultured in a minimum essential medium (MEM) (BasalMedia, Shanghai, China) containing 5.5 mM glucose, 10% fetal bovine serum (FBS), and 1% penicillin/STZ in a humidified atmosphere of 5% CO_2_ at 37 °C. HaCaT cells maintained in the normal-glucose MEM (5.5 mM) served as the Sham group. HaCaT cells cultured in the MEM with HG (50 mM) for 24 or 48 h were used as the control group. Then, HG-induced HaCaT cells were treated with NAR (0.12, 0.37, 1.1, and 3.3 μM) for 24 h.

Chloroquine (CQ) (Sangon Biotech Co., Ltd.) and 3-methyladenine (3-MA) (Aladdin, Shanghai, China) were employed to detect the effect of NAR on the autophagic flux. To verify the role of ERs in the wound healing-promoting effect of NAR, tamoxifen (TAM) (Solarbio, Beijing, China), an ERs modulator, was utilized.

### 2.7 Plasmids and small interfering RNA (siRNA) transient transfection

The plasmids for plasmid encoding enhanced green fluorescent protein (pEGFP)-C1-Parkin (kindly provided by Prof. Jun Zhou from Nankai University), pGU6-green fluorescent protein (GFP)-Neo (GenePharma, Shanghai, China), pGU6-GFP-short hairpin RNA targeting *PRKN* (sh*PRKN*) constructed based on pGU6/GFP/Neo-short hairpin RNA negative control (shNC) or the siRNA targeting Parkin (Sangon Biotech Co., Ltd.) were transfected into HaCaT cells by Lipofectamine 3000 reagent (Thermo Fisher Scientific Inc., Waltham, MA, USA) following the manual provided by the manufacturer when cells were grown to 70% confluency. The target sequences for siRNA against human Parkin were as follows: sense, 5′-CUUGGCUACUCCCUGCCUU-3′; antisense, 5′-AAGGCAGGGAGUAGCCAAG-3′.

### 2.8 Lentivirus (Lv) infection and stable cell line construction

Lv-mCherry-GFP-microtubule-associated proteins 1A/1B light chain 3C (LC3), Lv-mCherry-translocase of outer mitochondrial membrane 20 (Tomm20)-N10, and Lv-mitochondria-targeted monomeric Keima (mt-mKeima) were purchased from OBIO Co., Ltd. (Shanghai, China). HaCaT cells were sub-cultured at a ratio of 1:10 and maintained under 5% CO_2_ at 37 °C. After 24 h, the cells were infected with Lv for 48 h. Stable cell lines were selected by adding puromycin at final concentrations of 0.06, 0.18, 0.55, 1.67, and 5 μg/mL. The cells with a high positive rate were collected and cryopreserved at −80 °C for subsequent experiments.

### 2.9 Cell scratch assay

When HaCaT cells reached a confluent state, the cell culture monolayers were scratched with a pipette tip and rinsed with PBS to remove debris. An optical microscope (Olympus, Tokyo, Japan) was used to capture images in the same scratch position at 0, 3, 6, and 9 h after the scratch. The scratch area in each picture was quantified using ImageJ (NIH).

### 2.10 Cell apoptosis detection by flow cytometry

The apoptosis of HaCaT cells was measured using the annexin V-fluorescein isothiocyanate (FITC) apoptosis detection kit (Sangon Biotech Co., Ltd.) following the manufacturer's instructions. Briefly, the cells were digested using trypsin and re-suspended in the binding buffer containing annexin V-FITC dye and stained at RT for 15 min in darkness. After being washed with the binding buffer, the samples were re-suspended in the propidium iodide (PI) staining solution and immediately examined in the FITC and phycoerythrin channels using a flow cytometer (Agilent Technologies, Santa Clara, CA, USA). The data were analyzed using the NovoExpress software (Agilent Technologies).

### 2.11 Senescence-associated β-galactosidase (SA-β-gal) staining

HaCaT cells were seeded into 24-well plates, and senescent cells were detected with the SA-β-gal kit (Beyotime Biotechnology, Shanghai, China) according to the manufacturer's instructions. Briefly, cells were washed twice with PBS and fixed with the β-galactosidase staining solution at RT for 15 min. After being washed with PBS, cells were incubated with dye working solution overnight at 37 °C. Senescent cells exhibited a blue-stained cytoplasm, indicating a positive result. Three random fields of view were selected for each well, and images were captured using an optical microscope (Olympus). The SA-β-gal positive areas of each image were measured using ImageJ (NIH).

### 2.12 Determination of reactive oxygen species (ROS) production

Dihydroethidium (DHE) fluorescent probe (Aladdin) was utilized to detect intracellular superoxide production. The fluorescence microscope method for DHE staining was performed as described previously with some modifications. Briefly, HaCaT cells were loaded with a DHE probe (20 μM) at 37 °C for 30 min in darkness. Intracellular DHE fluorescence intensity was visualized using an inverted fluorescence microscope (Zeiss, Oberkochen, Germany). For enzyme labeling analysis, HaCaT cells were digested, collected, and labeled with a DHE probe at RT for 30 min in darkness. The DHE fluorescence signal was detected by a multimode microplate reader (Tecan, Morgan Hill, CA, USA). The fluorescence intensity at 370 nm indicates the amount of non-oxidized DHE, while the intensity at 535 nm reflects the amount of oxidized DHE. The 535 nm/370 nm ratio was quantified to assess ROS levels in each group.

Mitochondrial ROS (mtROS) production was measured using the MitoSOX Red mitochondrial superoxide indicator (Thermo Fisher Scientific Inc.) in live cells. Briefly, cells were incubated using the MitoSOX indicator at 37 °C in 5% CO_2_ for 30 min. After being rinsed twice with a preheated serum-free medium, the intracellular MitoSOX fluorescence signal was detected by an inverted fluorescence microscope (Zeiss).

### 2.13 Analysis of mitochondrial morphology

Mitochondrial morphology of HaCaT cells was visualized by staining with 200 nM MitoTracker orange (Thermo Fisher Scientific Inc.) at 37 °C for 30 min. Three image slices were collected using a confocal microscope (Zeiss) with a 550 nm argon laser in the “*z*-stack” mode. Mitochondrial analysis was performed using the ImageJ (NIH) mitochondrial analyzer plugin. The form factor (FF) is defined as (perimeter^2^/4π area) to represent mitochondrial complexity. If the mitochondrion is a sphere, its FF is defined as the minimum value of 1. The number of branches, branch length, and branch junctions of mitochondria reflect the health of the mitochondrial network.

### 2.14 Mitochondrial membrane potential (MMP) determination

MMP was determined using a 5,5′,6,6′-tetrachloro-1,1′,3,3′-tetraethyl-imidacarbocyanine iodide (JC-1) probe (MedChemExpress, Monmouth Junction, NJ, USA) according to the manufacturer's protocol. Briefly, HaCaT cells were labeled with JC-1 (2.5 μM) at 37 °C for 30 min, and then fluorescence was measured using an inverted fluorescence microscope (Zeiss). JC-1 aggregates (572 nm) represent mitochondria with normal membrane potential, while JC-1 monomers (525 nm) represent mitochondria with membrane potential depolarization.

### 2.15 Mito-Keima mitophagy assay

HaCaT cells stably expressing mito-mKeima were used to detect mitophagy. The fluorescence signals were captured by a confocal microscope (Zeiss) using an argon laser at the excitation wavelength of 550 nm (mt-mKeima at acidic pH).

### 2.16 Autophagic flux detection

HaCaT cells stably expressing mCherry-GFP-LC3 were utilized to detect the autophagic flux. After fixation with 4% PFA at RT for 20 min, cells were permeabilized with 0.5% Triton X-100 (Sangon Biotech Co., Ltd.) for 15 min. Subsequently, cells were incubated with 4% bovine serum albumin (BSA) (Neofroxx, Einhausen, Germany) at RT for 1 h. Nuclei were stained with Hoechst 33258 (Sigma-Aldirch) for 5 min. LC3 puncta were visualized and imaged by an inverted fluorescence microscope (Zeiss).

### 2.17 Mitochondrial isolation and purification

Mitochondria were isolated from HaCaT cells by the differential centrifugation method as described previously with slight modifications [44]. Briefly, HaCaT cells were digested, harvested using trypsin, and washed twice with pre-cooled PBS. Then, cell precipitates were re-suspended in the basal cell lysate, including Tris-HCl (25 mM) and NaCl (150 mM) supplemented with 1 mM phenylmethylsulfonyl fluoride (PMSF) (Yuanye, Shanghai, China). The cells were subsequently homogenized using a low-temperature tissue homogenizer (Servicebio, Wuhan, China) with 1-mm diameter zirconia grinding beads. The cell homogenates were centrifuged at 4 °C for 5 min at 720 *g*. Then, the supernatant was transferred to a new test tube and centrifuged at 4 °C for 20 min at 10,000 *g*. The supernatant was the cytoplasmic part, and the precipitates were the coarse-grained mitochondria. The resulting precipitates were re-suspended and centrifuged at 4 °C for further 10 min at 15,000 *g* to obtain a purer mitochondrial component.

### 2.18 Immunofluorescence assay

HaCaT cells were fixed with 4% PFA for 20 min, permeabilized by 0.5% Triton X-100, and blocked with 4% BSA for 1 h. Afterward, the cells were stained with the primary antibodies against Ki67 (Santa Cruz Biotechnology, Dallas, TX, USA), LC3 (MBL, Tokyo, Japan), and Tomm20 (Proteintech, Rosemont, IL, USA) in 4% BSA for 2 h at RT, followed by thrice washing with PBS. Alexa-labeled secondary antibodies (Jackson Immuno, West Grove, PA, USA) diluted in 4% BSA were used for immunostaining. Nuclei were stained with Hoechst 33258 (Sigma-Aldirch) for 5 min. Immunofluorescence images were captured using a confocal microscope (Zeiss).

### 2.19 Western blotting

HaCaT cells were lysed using 1× sodium dodecyl sulfate-polyacrylamide gel electrophoresis (SDS-PAGE) sample loading buffer, heated at 98 °C for 10 min, and centrifuged at 13,680 *g* for 3 min at RT. Equal amounts of the supernatant were subjected to SDS-PAGE and transferred onto a polyvinylidene fluoride (PVDF) membrane. After blocking with 5% fat-free milk in Tris-buffered saline containing 0.1% Tween 20, the PVDF membrane was incubated with primary antibodies against β-actin (Santa Cruz Biotechnology), β-tubulin (Biodragon, Suzhou, China), p-H2A histone family member X (p-γH2AX) (Bioss, Beijing, China), adenosine triphosphate (ATP) synthase F1 subunit alpha (ATP5F1A) (Sangon Biotech Co., Ltd.), Cav-1 (Sangon Biotech Co., Ltd.), cytochrome C oxidase subunit 4 (COX IV) (Proteintech), dynamin-1-like protein (DRP1) (Sangon Biotech Co., Ltd.), ERα (Proteintech), ERβ (Sangon Biotech Co., Ltd.), glyceraldehyde 3-phosphate dehydrogenase (GAPDH) (Proteintech), glutaredoxin 1 (GRX1) (Proteintech), histone H3 (Proteintech), heat shock protein 60 (HSP60) (Sangon Biotech Co., Ltd.), LaminB1 (Sangon Biotech Co., Ltd.), LC3 (MBL), mitofusin 2 (MFN2) (Proteintech), cytochrome C oxidase subunit 2 (MT-CO2) (Sangon Biotech Co., Ltd.), nicotinamide adenine dinucleotide (NADH) dehydrogenase (ubiquinone) iron-sulfur protein 4 (Ndufs4) (Sangon Biotech Co., Ltd.), nuclear factor-κB (NF-κB) (Proteintech), NQO1 (Sangon Biotech Co., Ltd.), nuclear respiratory factor 1 (NRF1) (Proteintech), cyclin-dependent kinase inhibitor 1A (P21) (Proteintech), sequestosome-1 (P62) (Proteintech), peroxisome proliferator-activated receptor gamma coactivator 1-alpha (PGC-1α) (Proteintech), PTEN-induced putative kinase 1 (PINK1) (Novus Biologicals, Littleton, CO, USA), p-NF-κB (S536) (Cell Signaling Technology), p-PINK1 (S228) (Thermo Fisher Scientific Inc.), succinate dehydrogenase complex flavoprotein subunit A (SDHA) (Proteintech), superoxide dismutase 1 (SOD1) (Proteintech), SOD2 (Proteintech), transcription factor A mitochondrial (TFAM) (Proteintech), translocase of inner mitochondrial membrane 23 (Tim23) (BD Biosciences, San Jose, CA, USA), Tomm20 (Proteintech), and voltage-dependent anion-selective channel protein 1 (VDAC1) (Sangon Biotech Co., Ltd.) overnight at 4 °C. Horseradish peroxidase-conjugated IgG (Invitrogen) and enhanced chemiluminescence (Biodragon) were applied to visualize the protein bands on a chemiluminescence imager (Clinx, Shanghai, China). The densitometric analysis of the strips was carried out using ImageJ (NIH).

### 2.20 Reverse transcription-quantitative polymerase chain reaction (RT-qPCR)

Total RNA was extracted from HaCaT cell lysate by a TransZol Up kit (TransGen, Beijing, China) following the manufacturer's instructions. First-strand complementary DNA (cDNA) was synthesized using the First Strand cDNA Synthesis Mix for qPCR (YEASEN, Shanghai, China). Quantitative analysis of cDNA was performed using an SYBR qPCR Mix (Novoprotein, Chengdu, China) on a real-time fluorescence quantitative PCR system (Bio-Rad, Hercules, CA, USA). Gene expression level was normalized to β-actin. Primer sequences used for RT-qPCR are listed in Table S1.

### 2.21 Cellular thermal shift assay (CETSA)

When 293T cells reached 80%–90% confluence, the culture medium was replaced with a fresh Dulbecco's modified Eagle medium (DMEM) (BasalMedia) containing 3.3 μM NAR. The cells were then incubated at 37 °C with 5% CO_2_ for 8 h. After incubation, basal cell lysate supplemented with PMSF was added, and cells were collected using a cell scraper. The cell suspensions from each group were aliquoted into eight PCR tubes. These tubes were subjected to a temperature gradient in a T100 thermal cycler (Bio-Rad), ranging from 37 to 62 °C for 4 min. Following heat treatment, the suspensions underwent three freeze-thaw cycles, alternating between liquid nitrogen and a 37 °C water bath. The cell lysates were centrifuged at 4 °C for 20 min at 20,000 *g*. The supernatant was transferred to a new microcentrifuge tube, mixed with 5× SDS-PAGE sample loading buffer, and heated at 98 °C for 10 min. The heated samples were subsequently used for Western blotting.

### 2.22 Luciferase reporter assay

The luciferase reporter assay, using the pER-TA-Luc plasmid (Beyotime Biotechnology) containing ERE sequences, was performed to assess ERs-mediated transcriptional regulation. 293T cells were seeded into six-well plates and cultured in a DMEM supplemented with 10% FBS for 24 h before transfection. When the cell convergence reached 70%, the cells were transfected with pER-TA-Luc plasmid. After 12–24 h of transfection, the cells were digested, collected, and seeded into a new six-well plate. When the cell convergence reached 70%–80%, they were treated with NAR (0.12, 0.37, 1.1, and 3.3 μM) or 17β-estradiol (E_2_) (100 nM) for 24 h. Cells were lysed using a harvest buffer, and equal volumes of the cell lysate were transferred to a 96-well plate. A reaction solution containing ATP and luciferin was added, followed by thorough mixing to initiate the luminescence reaction. Luciferase activity was measured using a multimode microplate reader (Tecan).

### 2.23 Statistical analysis

The results were expressed as the mean ± standard deviation (SD). Statistical differences were analyzed using one-way analysis of variance (ANOVA), two-way ANOVA, or Student's *t*-test through GraphPad Prism 8.0 software (GraphPad Software Inc., La Jolla, CA, USA). Differences were statistically significant when the *P*-value was less than 0.05.

## 3 Results

### 3.1 NAR significantly promoted the wound healing in DFU both *in vivo* at the epidermal basal layer and *in vitro*

**Fig. 1:**
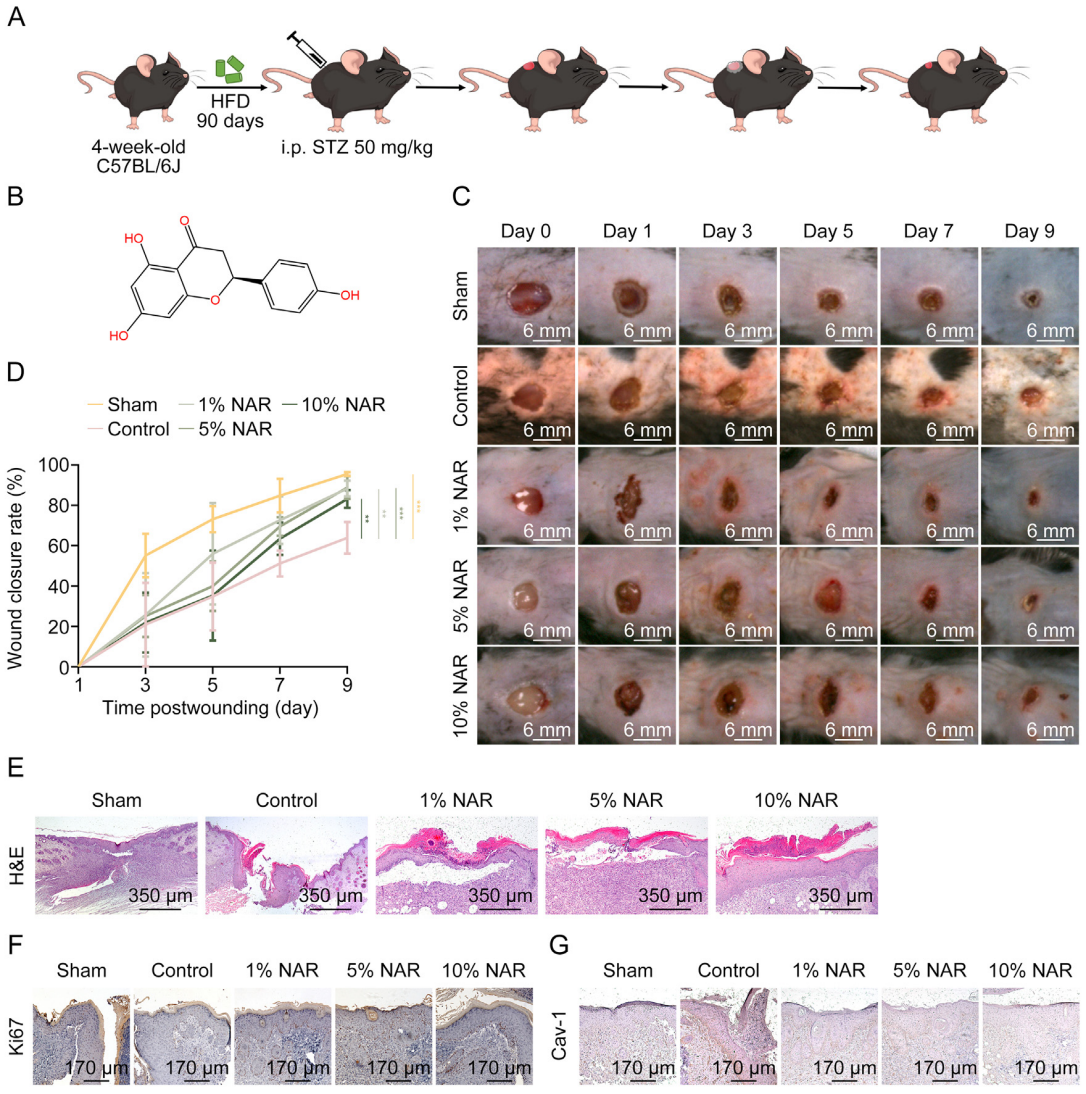
Naringenin (NAR) accelerates wound healing in diabetic foot ulcer (DFU) mice. (A) Flowchart of DFU murine model construction and treatment. A round wound with a diameter of 6 mm was made on the back of each mouse. NAR (1%, 5%, 10%) or blank ointments (BOs) were applied around the wounds for 10 consecutive days. (B) The chemical structure of NAR. (C) The representative images of the wound healing process of mice at days 0, 1, 3, 5, 7, and 9 post-puncture (*n* = 6). (D) Wound closure rates were calculated using images on days 1, 3, 5, 7, and 9 using ImageJ. (E) Representative images of histological assessment of gap closure in the wound epithelia of mice at day 9 post-puncture. (F, G) Representative immunohistochemistry (IHC) staining images of proliferation marker protein Ki67 and caveolin-1 (Cav-1) in wounds of mice at day 9 post-puncture. Data are presented as mean ± standard deviation (SD). ^∗∗^*P* < 0.01 and ^∗∗∗^*P* < 0.001, compared with the control group. HFD: high-fat diet; i.p.: intraperitoneal; STZ: streptomycin; H&E: hematoxylin and eosin.

DFU mouse models were established using male C57BL/6J mice following the method illustrated in Fig. 1A, and NAR (Fig. 1B) was applied topically. By the final day of treatment, the control group exhibited a larger wound area compared with the Sham group. NAR significantly enhanced DFU wound healing (Figs. 1C and D). The wound closure rates for the 1%, 5%, and 10% NAR groups were 91.3%, 91.4%, and 86.4%, respectively, all exceeding those in the control group (67.4%). Notably, at the mid-stage of wound healing (day 5), 1% NAR demonstrated a significantly stronger effect on wound closure than 5% or 10% NAR. By day 7, the wound healing rate in the 5% NAR group elevated, approaching that in the 1% NAR group. At the late stage of wound healing (day 9), 5% NAR exhibited a slightly higher wound closure rate than 1% NAR (Fig. 1D). However, the differences in therapeutic effects between adjacent NAR concentrations were not highly significant (Fig. 1D). Histological analysis of wound epithelia confirmed that wound closure was significantly faster in the NAR-treated groups compared with the control group (Fig. 1E). Furthermore, Ki67 expression level in the basal layer of the epidermis, which was nearly undetectable in mice of the control group, was restored by NAR treatment (Fig. 1F). Cav-1 expression level, a key regulator of cellular senescence that is overexpressed in chronic DFU wounds [45,46], was markedly elevated in the epidermal basal layer of the wound margins in the control group. However, NAR treatment reduced the number of Cav-1-positive cells dose-dependently (Fig. 1G).

**Fig. 2:**
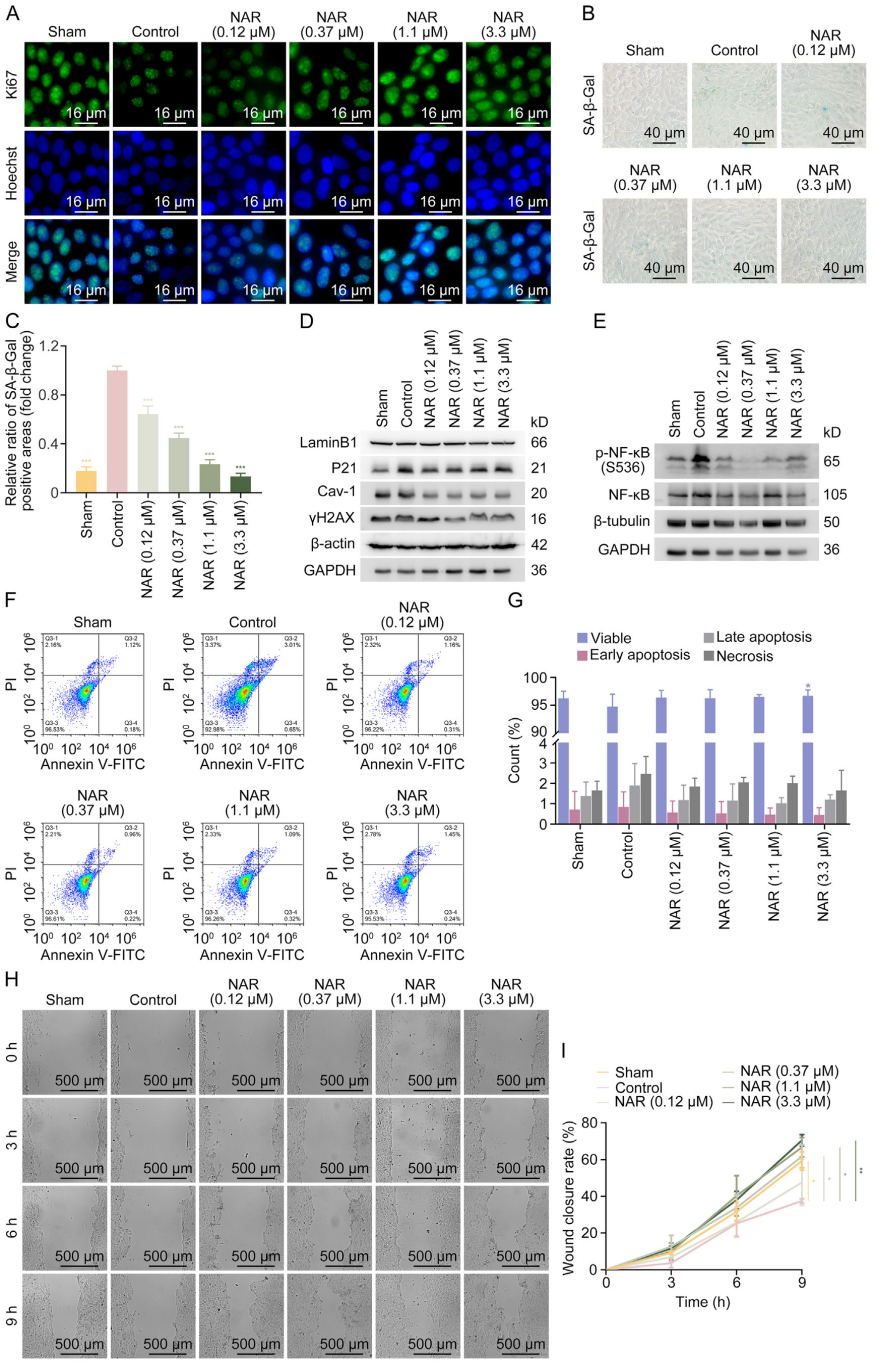
Naringenin (NAR) alleviates high glucose (HG)-induced keratinocyte dysfunction. HaCaT cells were treated with NAR (0.12, 0.37, 1.1, and 3.3 μM) for 24 h after being induced with HG (50 mM). (A) Immunofluorescence staining was used to detect the proliferation marker protein Ki67 expression in HaCaT cells. (B, C) The senescence of HaCaT cells was detected by senescence-associated β-galactosidase (SA-β-gal) staining (B) and quantification analysis (C). (D) The expression levels of senescence-related proteins, including LaminB1, cyclin-dependent kinase inhibitor 1A (P21), caveolin-1 (Cav-1), and phospho-H2A histone family member X (γH2AX) in HaCaT cells were measured by Western blotting. (E) The protein levels of nuclear factor-κB (NF-κB) and -NF-κB (S536) in HaCaT cells were measured by Western blotting. (F, G) The apoptotic rates of HaCaT cells were determined by flow cytometry (F) and quantification analysis (G). Two-way analysis of variance was performed for statistical analysis. (H) Representative images of the cell scratch assay in HaCaT cells. (I) The wound closure rate was calculated by measuring the scratch area using ImageJ. Data are presented as mean ± standard deviation (SD). ^∗^*P* < 0.05, ^∗∗^*P* < 0.01, and ^∗∗∗^*P* < 0.001, compared with the control group. GAPDH: glyceraldehyde 3-phosphate dehydrogenase; PI: propidium iodide; FITC: fluorescein isothiocyanate.

To further confirm NAR's effects on the basal layer of the epidermis in DFU, HG-induced human immortalized keratinocytes (HaCaT cells) were treated with NAR. While Ki67 expression level was significantly reduced in HG-induced HaCaT cells, NAR treatment restored its expression level, and the most notable effect was found at 1.1 μM (Fig. 2A). Additionally, SA-β-Gal staining revealed that NAR significantly decreased the accumulation of HG-induced senescent cells (Figs. 2B and C). Consistently, the HG-upregulated expression levels of senescence-related proteins, including LaminB1, P21, Cav-1, and γH2AX, were attenuated by NAR, suggesting that cell senescence was alleviated by NAR (Figs. 2D and S2A–D). Meanwhile, NAR inhibited the phosphorylation of NF-κB at S536 induced by HG (Figs. 2E and S2E). NAR rescued the necrosis and late apoptosis of HaCaT cells and inhibited early apoptosis induced by HG (Figs. 2F and G). As illustrated in Figs. 2H and I, HG significantly decreased the migratory ability of HaCaT cells as determined by the scratch assay, which was rescued by NAR. These results indicated that NAR could accelerate the wound healing of DFU *in vivo* and *in vitro* at the epidermal basal layer.

### 3.2 NAR attenuated oxidative stress and enhanced autophagic flux in DFU

**Fig. 3:**
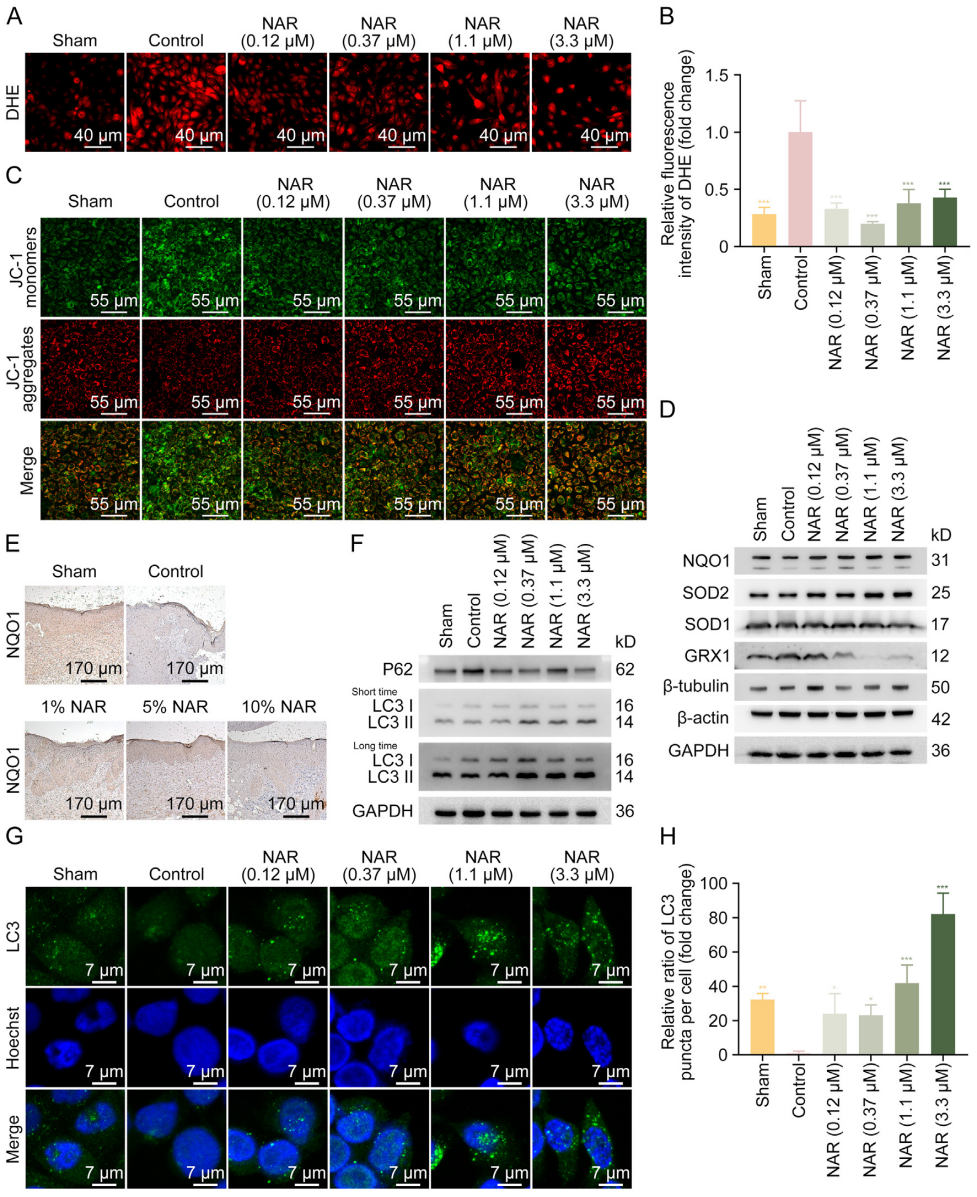
Naringenin (NAR) alleviates oxidative stress and inhibition of autophagy induced by high glucose (HG). (A) HaCaT cells were treated with NAR (0.12, 0.37, 1.1, and 3.3 μM) for 24 h after being induced with HG (50 mM) for 24 h. Then, HaCaT cells were incubated with dihydroethidium (DHE) probes for 30 min, and the reactive oxygen species (ROS) levels in each group were detected using inverted fluorescence microscope. (B) HaCaT cells were incubated with DHE probes for 30 min, and the fluorescence intensity of DHE at different excitation wavelengths was subsequently detected using a multifunctional microplate detection platform. The 535 nm/370 nm ratio was quantified to assess ROS levels. (C) HaCaT cells were incubated with 5,5′,6,6′-tetrachloro-1,1′,3,3′-tetraethyl-imidacarbocyanine iodide (JC-1) dye for 30 min, and the JC-1 fluorescence was then examined using inverted fluorescence microscope. JC-1 aggregates (red) represent mitochondria with normal membrane potential, while JC-1 monomers (green) represent mitochondria with membrane potential depolarization. (D) The levels of oxidative stress-related proteins, including nicotinamide adenine dinucleotide phosphate (NAD(P)H):quinone oxidoreductase 1 (NQO1), superoxide dismutase 2 (SOD2), SOD1, and glutaredoxin 1 (GRX1), in HaCaT cells were measured by Western blotting. (E) Representative immunohistochemistry (IHC) staining images of NQO1 in wounds of mice at day 9 post-puncture. (F) The levels of sequestosome-1 (P62) and microtubule-associated proteins 1A/1B light chain 3C (LC3) in HaCaT cells were detected by Western blotting. (G) HaCaT cells were immunostained with antibody against LC3 and visualized using confocal microscope. (H) The LC3 puncta in the cells were quantified by ImageJ. Data are presented as mean ± standard deviation (SD). ^∗^*P* < 0.05, ^∗∗^*P* < 0.01, and ^∗∗∗^*P* < 0.001, compared with the control group. GAPDH: glyceraldehyde 3-phosphate dehydrogenase.

As shown by DHE staining, HG-induced ROS accumulation was significantly reduced by NAR (Figs. 3A and B). Additionally, HG disrupted MMP, as indicated by an increased intracellular accumulation of JC-1 monomers (green) and a reduction of JC-1 aggregates (red). NAR treatment at 0.12, 0.37, 1.1, and 3.3 μM effectively reversed these changes (Fig. 3C). Consistently, NAR upregulated the expression levels of NQO1, SOD2, and SOD1, while downregulated GRX1 expression level (Figs. 3D and S2F–I). Furthermore, IHC staining of diabetic mouse wounds revealed that NAR restored NQO1 expression level in the epidermis (Fig. 3E). Collectively, these findings indicate that NAR alleviates oxidative stress in DFU by enhancing the expression levels of antioxidative proteins.

As autophagy plays a protective role against oxidative stress, the effects of NAR on autophagy in HG-induced HaCaT cells were examined. LC3II was decreased in HaCaT cells exposed to HG, accompanied by the increased expression of P62. However, NAR treatment enhanced LC3 lipidation and decreased P62 levels (Figs. 3F, S2J, and S2K). Consistently, NAR increased the number of LC3 puncta in HG-treated HaCaT cells (Figs. 3G and H). As autophagy is a dynamic and continuous process, HaCaT cells stably expressing mCherry-GFP-LC3 were further treated with autophagic flux inhibitors, CQ or 3-MA, to evaluate the effects of NAR on autophagic flux. As illustrated in Fig. S3A, HG treatment decreased the number of intracellular LC3 puncta, and nearly no red puncta were observed. NAR increased both yellow puncta (indicating autophagosomes, containing both red and green fluorescence) and red-only puncta (representing autolysosomes) in HG-treated HaCaT cells, an effect that was blocked by 3-MA. CQ further enhanced the effect of NAR on the accumulation of intracellular yellow LC3 puncta, while it inhibited the promotion of red-only puncta (autolysosomes) by NAR. Similarly, Western blotting analysis revealed that NAR increased the LC3II protein levels in HG-induced HaCaT cells, which was further amplified by CQ and abolished by 3-MA (Figs. S3B and C). These results indicate that NAR could enhance autophagic flux by promoting the formation of autophagosomes and facilitating the degradation of autolysosomes under HG stress.

### 3.3 NAR maintained the balance of MQC in DFU

**Fig. 4:**
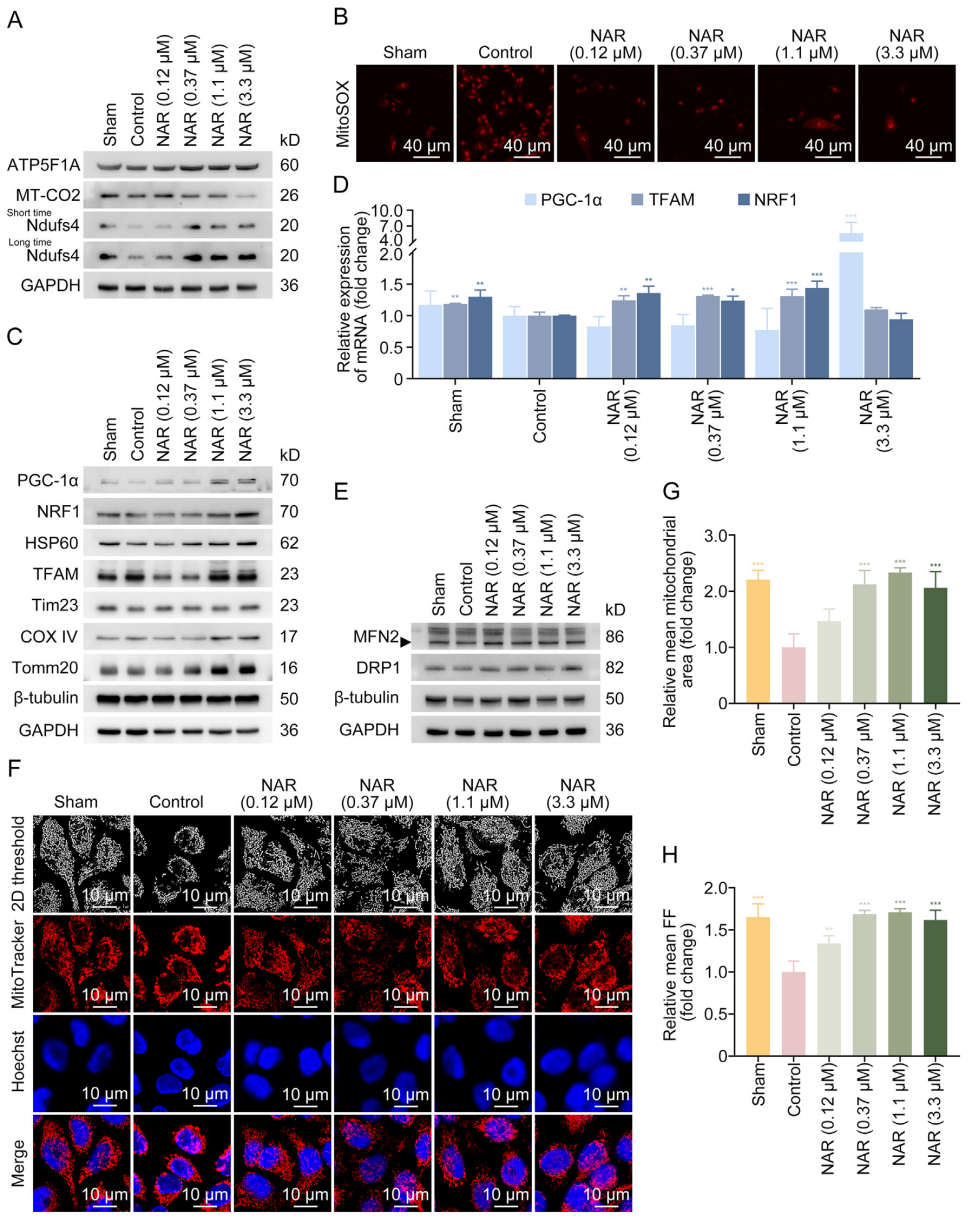
Naringenin (NAR) maintains mitochondrial homeostasis under high glucose (HG) stress. (A) HaCaT cells exposed to HG (50 mM) for 24 h were treated with NAR (0.12, 0.37, 1.1, and 3.3 μM) for 24 h. The expression levels of mitochondrial function-related proteins, including adenosine triphosphate (ATP) synthase F1 subunit alpha (ATP5F1A), cytochrome C oxidase subunit 2 (MT-CO2), and nicotinamide adenine dinucleotide (NADH) dehydrogenase (ubiquinone) iron-sulfur protein 4 (Ndufs4), in HaCaT cells were measured by Western blotting. (B) HaCaT cells were incubated with MitoSOX probes for 30 min to detect mitochondrial superoxide levels, followed by inverted fluorescence microscope. (C) The expression levels of mitochondrial biogenesis-related proteins, including peroxisome proliferator-activated receptor gamma coactivator 1-alpha (PGC-1α), nuclear respiratory factor 1 (NRF1), heat shock protein 60 (HSP60), transcription factor A, mitochondrial (TFAM), translocase of inner mitochondrial membrane 23 (Tim23), cytochrome C oxidase subunit 4 (COX IV), and translocase of outer mitochondrial membrane 20 (Tomm20), in HaCaT cells were measured by Western blotting. (D) The messenger RNA (mRNA) levels of PGC-1α, TFAM, and NRF1 were measured by reverse transcription-quantitative polymerase chain reaction (RT-qPCR). (E) The protein levels of mitofusin 2 (MFN2) and dynamin-1-like protein (DRP1) in HaCaT cells were determined by Western blotting. The arrowhead indicates the band of MFN2. (F) Representative fluorescence images of mitochondrial morphology of HaCaT cells stained with MitoTracker as observed by confocal microscope and two-dimensional (2D) threshold images using ImageJ. (G, H) Analysis of the mean mitochondrial area (G) and mean form factor (FF) (H) from Fig. 4F using ImageJ. Data are presented as mean ± standard deviation (SD). ^∗^*P* < 0.05, ^∗∗^*P* < 0.01, and ^∗∗∗^*P* < 0.001, compared with the control group. GAPDH: glyceraldehyde 3-phosphate dehydrogenase.

**Fig. 5:**
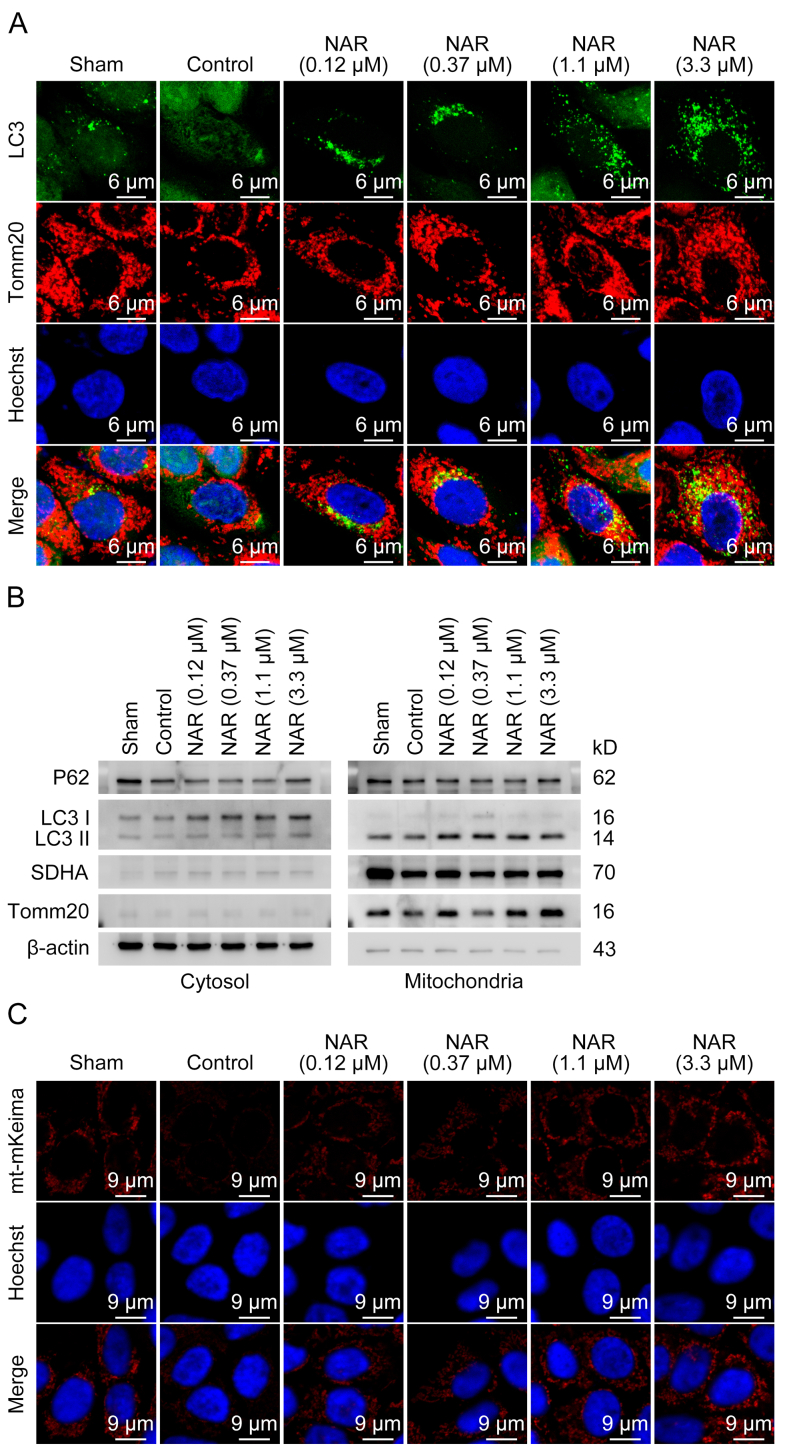
Naringenin (NAR) promotes the mitophagy of HaCaT cells under high glucose (HG) conditions. (A) HaCaT cells stably expressing mCherry-green fluorescent protein (GFP)-microtubule-associated proteins 1A/1B light chain 3C (LC3) were stained with anti-translocase of outer mitochondrial membrane 20 (Tomm20) antibody and imaged using confocal microscope to examine the mitochondrial localization of LC3. (B) Representative immunoblots of sequestosome-1 (P62) and LC3 from cytoplasmic and mitochondrial fractions of HaCaT cells. Tomm20 and succinate dehydrogenase complex flavoprotein subunit A (SDHA) were used as mitochondrial markers, while β-actin was used as a cytosolic marker. (C) HaCaT cells stably expressing mitochondria-targeted monomeric Keima (mt-mKeima) were treated with NAR (0.12, 0.37, 1.1, and 3.3 μM) for 24 h after being induced with HG (50 mM) for 24 h. The fluorescence was detected by confocal microscope. The red fluorescence of mt-mKeima corresponds to mitochondria located in lysosomes.

Since the imbalance in MQC is a major contributor to oxidative stress and NAR alleviates oxidative stress, the effects of NAR on MQC in DFU were further determined. As detected by Western blotting, NAR significantly elevated mitochondrial function-related protein levels of ATP5F1A (complex V) and Ndufs4 (complex I), and slightly upregulated MT-CO2 (complex IV) level at 0.12 μM, indicating that NAR maintained normal mitochondrial function (Figs. 4A and S4A–C). Furthermore, NAR dose-dependently inhibited HG-induced mtROS accumulation (Fig. 4B). It was revealed that HG reduced the levels of PGC-1α, NRF1, HSP60, and Tim23, which was reversed by NAR (Figs. 4C and S4D–G). HG did not impact the protein levels of Tomm20, COXIV, and TFAM (Figs. 4C and S4H–J). Consistently, NAR enhanced the transcription of PGC-1α, TFAM, and NRF1 (Fig. 4D). NAR rescued the expression levels of MFN2 and DRP1 inhibited by HG, indicating that NAR promoted mitochondrial dynamics (Figs. 4E, S4K, and S4L). To visualize the mitochondrial morphology, MitoTracker staining and two-dimensional (2D) threshold analysis of fluorescence images were performed. The mitochondrial network in the Sham group appeared as normal long, tubular mitochondria (Fig. 4F). HG induced extensive mitochondrial fragmentation and swelling, transforming the mitochondria from long tubular structures to spherical shapes, and decreased the number of mitochondria (Figs. 4F–H and S4M–O). Notably, NAR maintained mitochondrial network complexity and increased the number of mitochondrial branches and branch junctions (Figs. 4F–H and S4M–O). The effects of NAR on mitophagy were assessed by evaluating the co-localization of LC3 and Tomm20. HG significantly decreased the co-localization of LC3 and Tomm20, resulting in an abnormal mitochondrial network morphology. NAR promoted the colocalization of LC3 and Tomm20, suggesting the recovery of mitophagy (Fig. 5A). Additionally, HG inhibited the binding of P62 and LC3II to mitochondria, which was reversed by NAR, suggesting that NAR restored HG-inhibited mitophagy (Fig. 5B). To further confirm the occurrence of mitophagy, HaCaT cells stably expressing mt-mKeima, a pH-sensitive fluorescent protein targeting mitochondria, were established. During mitophagy, as mitochondria were transported from the cytoplasm to the lysosome, mt-mKeima exhibited a shift in the excitation spectrum peak and emitted red fluorescence. As depicted in Fig. 5C, NAR significantly increased red spots in the cytoplasm, indicating that NAR enhanced the movement of mitochondria to the lysosome. The findings suggested that NAR treatment could maintain the balance of MQC and protect mitochondrial function against damage caused by HG stress.

### 3.4 NAR alleviated oxidative stress and maintained mitochondria function by promoting Parkin-mediated mitophagy

**Fig. 6:**
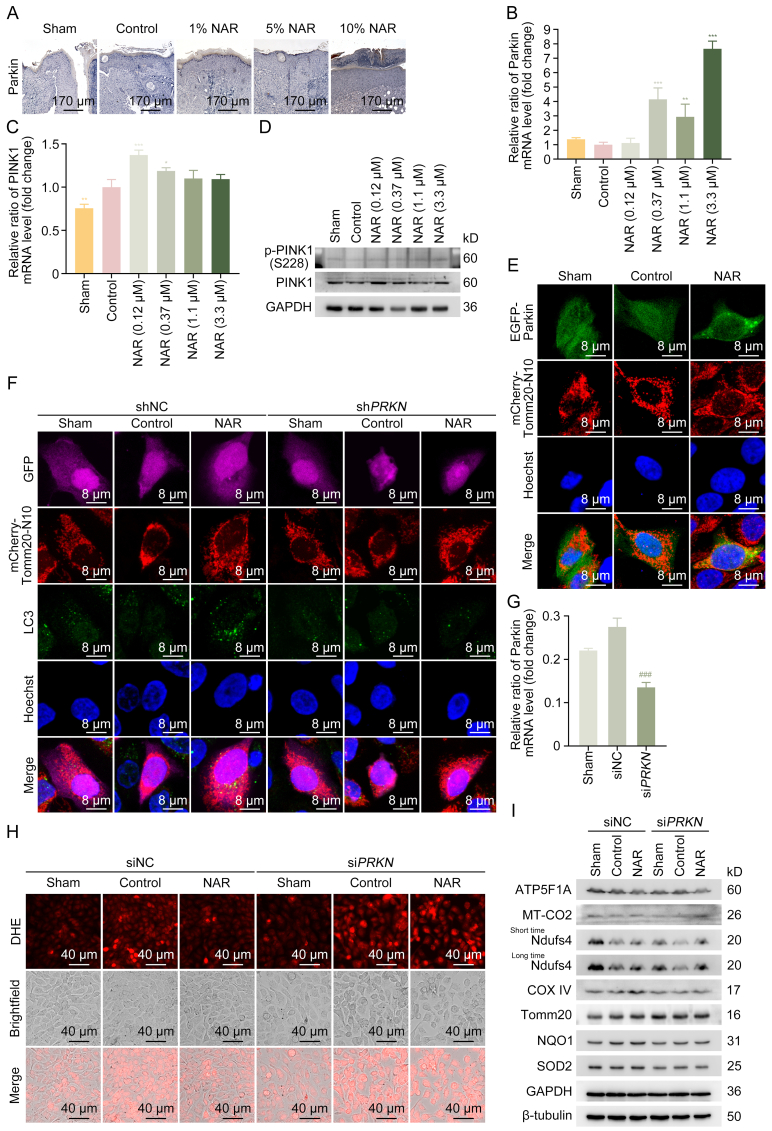
E3 ubiquitin-protein ligase parkin (Parkin/PRKN/Prkn)-mediated mitophagy is essential for the protective effects of naringenin (NAR) under high glucose (HG) conditions. (A) Representative immunohistochemistry (IHC) staining images of Parkin in wounds of mice at day 9 post-puncture. (B) HaCaT cells exposed to HG (50 mM) for 24 h were treated with NAR (0.12, 0.37, 1.1, and 3.3 μM) for a further 24 h. The messenger RNA (mRNA) level of Parkin was measured by reverse transcription-quantitative polymerase chain reaction (RT-qPCR). (C) The mRNA level of PTEN-induced putative kinase 1 (PINK1) was measured by RT-qPCR. (D) The expression levels of PINK1 and p-PINK1 (S228) in HaCaT cells were measured by Western blotting. (E) HaCaT cells stably expressing mCherry-translocase of outer mitochondrial membrane 20 (Tomm20)-N10 were transfected with EGFP-Parkin. After 24 h of transfection, cells were induced with HG (50 mM) for 24 h and then treated with NAR (3.3 μM) for another 24 h. Cells were visualized and imaged by confocal microscope to evaluate the mitochondrial localization of Parkin. (F) HaCaT cells stably expressing mCherry-Tomm20-N10 were transfected with a control vector or green fluorescent protein (GFP)-short hairpin RNA targeting *PRKN* (sh*PRKN*). After 24 h of transfection, cells were induced with HG (50 mM) for 24 h, followed by treatment with NAR (3.3 μM) for another 24 h. Then, cells were immunostained with anti-microtubule-associated proteins 1A/1B light chain 3C (LC3) and visualized by confocal microscope to evaluate the mitochondrial localization of LC3. (G) Parkin deficiency mediated by small interfering RNA (siRNA) in HaCaT cells was confirmed by RT-qPCR. (H) After 24 h of transfection with negative control siRNA (siNC) or si*PRKN*, HaCaT cells were treated with NAR (3.3 μM) for 24 h after being induced with HG (50 mM) for 24 h. Then, the cells were incubated with dihydroethidium (DHE) probes for 30 min to detect reactive oxygen species (ROS) levels, followed by inverted fluorescence microscope imaging. (I) The protein levels of adenosine triphosphate (ATP) synthase F1 subunit alpha (ATP5F1A), cytochrome C oxidase subunit 2 (MT-CO2), nicotinamide adenine dinucleotide (NADH) dehydrogenase (ubiquinone) iron-sulfur protein 4 (Ndufs4), cytochrome C oxidase subunit 4 (COX IV), Tomm20, nicotinamide adenine dinucleotide phosphate (NAD(P)H):quinone oxidoreductase 1 (NQO1), and superoxide dismutase 2 (SOD2) in HaCaT cells were detected by Western blotting. Data are presented as mean ± standard deviation (SD). ^∗^*P* < 0.05, ^∗∗^*P* < 0.01, and ^∗∗∗^*P* < 0.001, compared with the control group; ^###^*P* < 0.001 compared with the siNC group. GAPDH: glyceraldehyde 3-phosphate dehydrogenase; shNC: short hairpin RNA negative control.

Due to the crucial role of Parkin in mitochondrial homeostasis, the effects of NAR on Parkin were investigated. Firstly, the effects of NAR on Parkin expression were examined. The results from the Mouse Genome Informatics database indicated that the abundance of Parkin RNA expression in mouse skin tissue was either moderate or absent [47,48]. Consistently, the Parkin-positive signal in the basal layer of the skin from mice in the Sham group was weak, indicating a low basal expression level of Parkin (Fig. 6A). In contrast to the Sham group, the expression level of Parkin was further reduced in the epidermal basal layer in the control group, while NAR restored the Parkin expression level (Fig. 6A). NAR significantly enhanced the transcription of Parkin in HG-induced HaCaT cells (Fig. 6B). Consistently, NAR increased the messenger RNA (mRNA) level of PINK1 in HG-induced HaCaT cells (Fig. 6C). NAR further enhanced the autophosphorylation of PINK1 dose-dependently and increased PINK1 protein level at low concentrations (Figs. 6D and S5). HaCaT cells stably expressing mCherry-Tomm20-N10 were employed to examine the effects of NAR on the co-localization of Parkin and mitochondria. As illustrated in Fig. 6E, NAR markedly promoted the translocation of Parkin to mitochondria, indicating that NAR enhanced mitophagy. HG decreased the co-localization of LC3 and Tomm20, which was reversed by NAR. However, the effect of NAR on the co-localization of LC3 and Tomm20 was abolished by knocking down Parkin (Fig. 6F). These results suggested that NAR could promote Parkin-dependent mitophagy.

Parkin was further knocked down in HaCaT cells using siRNA, achieving satisfactory knockdown efficiency as confirmed by RT-qPCR (Fig. 6G). NAR alleviated oxidative stress, as indicated by DHE staining, an effect that was mitigated by Parkin knockdown (Fig. 6H). In the Sham group, Parkin deficiency alone reduced the expression levels of mitochondrial function-related proteins, including ATP5F1A, MT-CO2, and Ndufs4, suggesting that Parkin deficiency impaired mitochondrial function (Figs. 6I and S6A–C). Parkin deficiency abolished the upregulation of mitochondrial function-related proteins (Ndufs4 and COX IV) by NAR in HG-induced HaCaT cells (Figs. 6I, S6C, and S6D). Additionally, NAR not only failed to reduce, whereas increased the level of the mitochondrial protein Tomm20 in Parkin-deficient HaCaT cells, suggesting that NAR could not effectively promote mitophagy in the absence of Parkin (Figs. 6I and S6E). Additionally, NAR upregulated the levels of antioxidant enzymes NQO1 and SOD2 upon HG stimulation, which were abolished with Parkin knockdown (Figs. 6I, S6F, and S6G). These findings substantiated that Parkin-mediated mitophagy is critical for NAR to protect HaCaT cells from HG-induced oxidative stress and mitochondrial dysfunction.

### 3.5 The healing of NAR on DFU was abolished in *Prkn*^−/−^ mice

**Fig. 7:**
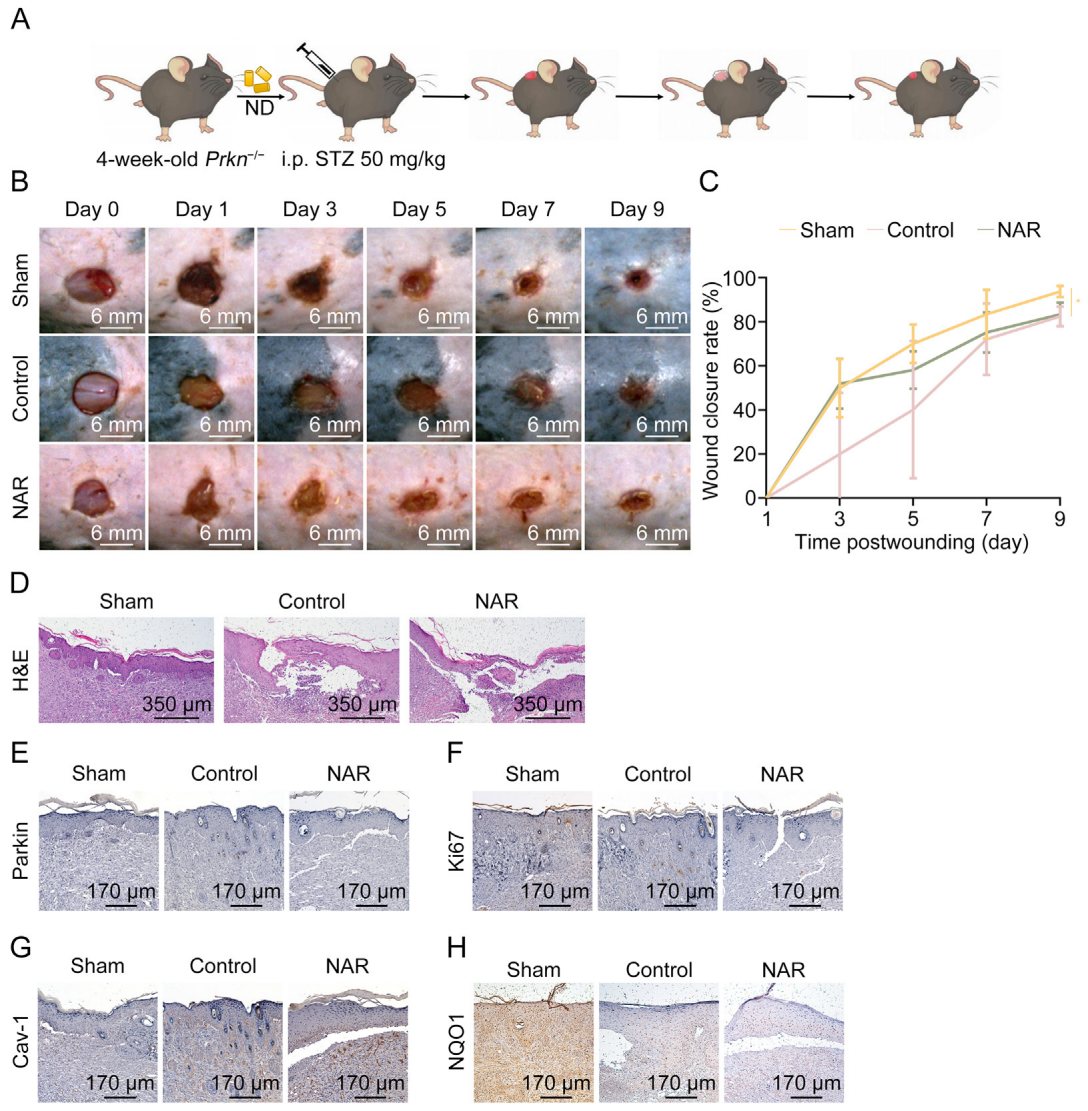
Naringenin (NAR)'s effects on diabetic wound healing are abolished in E3 ubiquitin-protein ligase parkin (Parkin/PRKN/Prkn) deficiency mice. (A) Flowchart of *Prkn* knockout (*Prkn*^*−*/−^) diabetic foot ulcer (DFU) murine model construction and treatment. The four-week-old male *Prkn*^*−*/−^ mice were utilized to confirm the role of Parkin in NAR's effects on DFU. After genotyping, the mice were intraperitoneally injected with streptozotocin (STZ) at a dose of 50 mg/kg for five consecutive days. Circular wounds of 6-mm diameter on the back of mice with fasting blood glucose (FBG) level above 200 mg/dL were created by a skin sampler. Blank or 5% NAR ointment was applied around the wounds for 10 consecutive days after injury. Each group of mice was maintained on a normal chow diet (CD) throughout the experiment. (B) Representative images of the skin wounds of *Prkn*^*−*/−^ mice on days 0, 1, 3, 5, 7, and 9 post-puncture (*n* = 5). (C) Wound closure rates in Fig. 7B were calculated using images captured on days 1, 3, 5, 7, and 9 through ImageJ. (D) Representative images of histological assessment of gap closure in the wound epithelia of *Prkn*^*−*/−^ mice on day 9 post-puncture. (E–H) Representative immunohistochemistry (IHC) staining images of Parkin (E), proliferation marker protein Ki67 (F), caveolin-1 (Cav-1) (G), and nicotinamide adenine dinucleotide phosphate (NAD(P)H):quinone oxidoreductase 1 (NQO1) (H) in the wound of *Prkn*^*−*/−^ mice on day 9 post-puncture. Data are presented as mean ± standard deviation (SD). ^∗^*P* < 0.05, compared with the control group. ND: normal diet; i.p.: intraperitoneal; H&E: hematoxylin and eosin.

To confirm the role of Parkin in NAR's healing efficacy in DFU, *Prkn*^*−*/−^ mice were generated and a DFU mouse model was established (Fig. 7A). As illustrated in Figs. 7B–D, the NAR and control groups showed similar delayed wound healing compared with the Sham group. In other words, Parkin deficiency could eliminate the healing effects of NAR on DFU. IHC results confirmed that NAR did not affect Parkin expression level in *Prkn*^*−*/−^ mice (Fig. 7E). Consistently, NAR did not upregulate Ki67 level in the epidermal basal layer in the control group, indicating that NAR's effects were eliminated (Fig. 7F). In the absence of Parkin, NAR could not prevent the accumulation of Cav-1 in the skin of mice in the control group (Fig. 7G). The antioxidative efficacy of NAR, as indicated by the enhanced expression level of NQO1, was also impaired in the absence of Parkin (Fig. 7H). These *in vivo* data highlighted that the effects of NAR on DFU are dependent on Parkin.

### 3.6 NAR maintained mitochondrial homeostasis and mitigated oxidative stress via binding to ERα

**Fig. 8:**
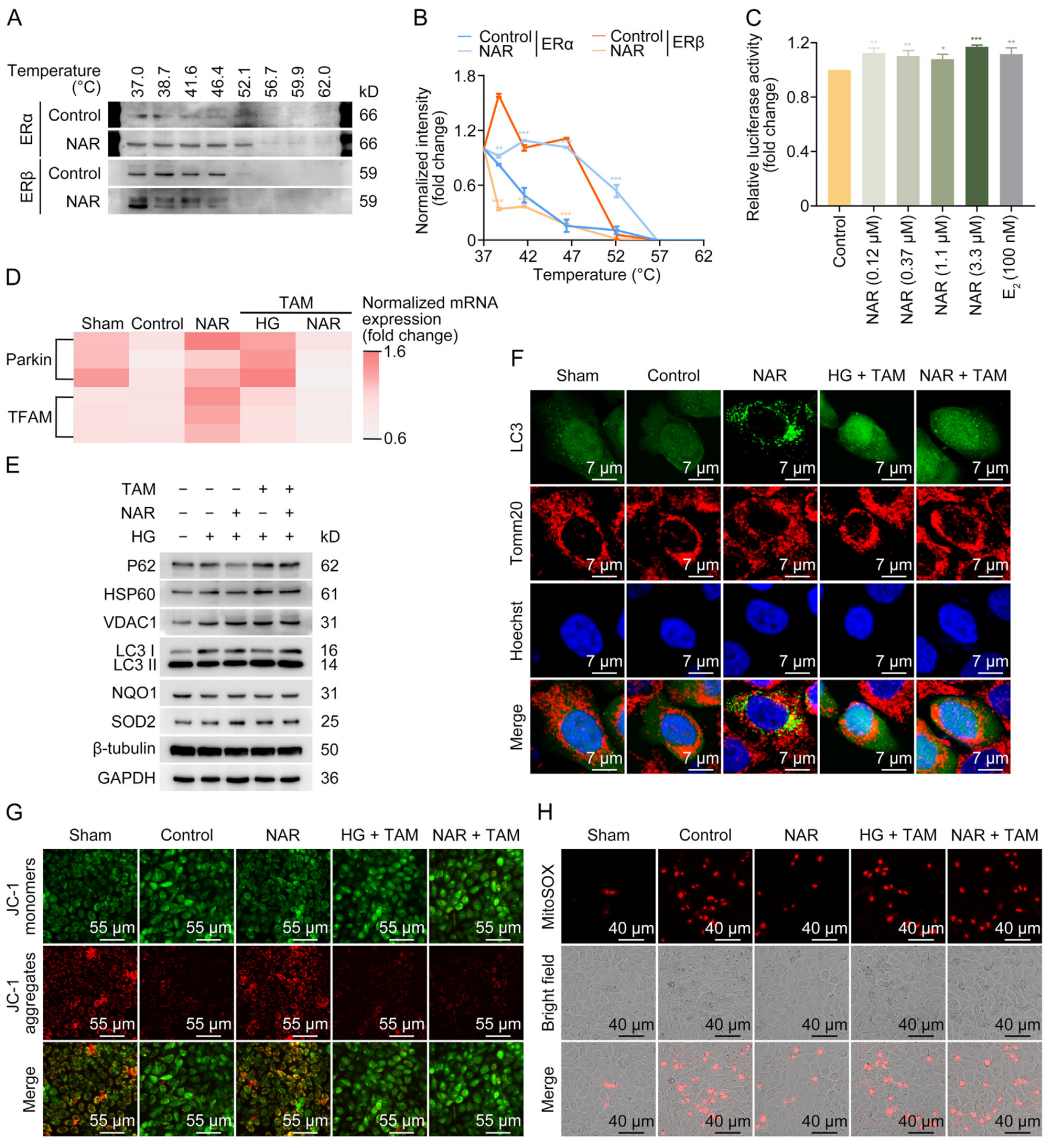
Naringenin (NAR) alleviates high glucose (HG)-induced damage of HaCaT cells via estrogen receptor alpha (ERα). (A, B) 293T cells were treated with NAR (3.3 μM) for 8 h. Cells were collected and incubated at different temperatures for 4 min. Cell lysates were used to detect the thermal stability of ERα and ERβ by Western blotting. Illustrating representative cellular thermal shift assay (CETSA) blots (A) and statistical analysis of densitometric measurements (B) of ERα and ERβ. (C) 293T cells were transfected with pER-TA-Luc plasmid encoding estrogen response element (ERE) and then treated with NAR (0.12, 0.37, 1.1, and 3.3 μM) or 17β-estradiol (E_2_) (100 nM) for 24 h. The luciferase activity was measured. (D) Quantitative statistical thermogram analysis of messenger RNA (mRNA) levels of E3 ubiquitin-protein ligase parkin (Parkin/PRKN/Prkn) and transcription factor A, mitochondrial (TFAM) in HaCaT cells. (E) The expression levels of sequestosome-1 (P62), heat shock protein 60 (HSP60), voltage-dependent anion-selective channel protein 1 (VDAC1), microtubule-associated proteins 1A/1B light chain 3C (LC3), nicotinamide adenine dinucleotide phosphate (NAD(P)H):quinone oxidoreductase 1 (NQO1), and superoxide dismutase 2 (SOD2) in HaCaT cells were determined using Western blotting. (F) HaCaT cells stably expressing mCherry-GFP-LC3 were exposed to HG (50 mM) for 24 h, followed by intervention with NAR (3.3 μM) and tamoxifen (TAM) (15 μM) alone or in combination for 24 h. Then, cells were immunostained with anti-translocase of outer mitochondrial membrane 20 (Tomm20) and visualized by confocal microscope to evaluate the mitochondrial localization of LC3. (G) HaCaT cells were incubated with 5,5′,6,6′-tetrachloro-1,1′,3,3′-tetraethyl-imidacarbocyanine iodide (JC-1) dye for 30 min. Then, the JC-1 fluorescence was examined using inverted fluorescence microscope. JC-1 aggregates (red) represent mitochondria with normal membrane potential, while JC-1 monomers (green) represent mitochondria with membrane potential depolarization. (H) HaCaT cells were incubated with MitoSOX probes for 30 min to detect mitochondrial superoxide levels, followed by inverted fluorescence microscope imaging. Data are presented as mean ± standard deviation (SD). ^∗^*P* < 0.05, ^∗∗^*P* < 0.01, and ^∗∗∗^*P* < 0.001, compared with the control group. GAPDH: glyceraldehyde 3-phosphate dehydrogenase.

Given that NAR plays a beneficial role through ERs and ERs regulate wound healing in diabetic mice, the impact of ERs on NAR-mediated wound healing in HaCaT cells was then assessed. The CETSA was employed to corroborate whether NAR could directly bind to ERs. Compared with the control group, more ERα was retained in NAR-treated cells at the same temperature, indicating that NAR enhanced the thermal stability of ERα and supported the direct interaction between NAR and ERα (Figs. 8A and B). However, the thermal stability of ERβ was not improved in NAR-treated cells, making it elusive whether there was an interaction between NAR and ERβ (Figs. 8A and B). ERα is a member of the nuclear receptor family, which translocates to the nucleus and acts as a transcription factor to regulate gene expression. A luciferase reporter plasmid containing ERE sequences was employed to detect whether NAR could directly influence the transcriptional regulation of ERα. The results indicated that compared with the control group, the fluorescence intensities of luciferin in NAR groups were significantly enhanced, indicating that NAR upregulated the relative activity of luciferase. Notably, the upregulation effect of NAR was similar to E_2_ (Fig. 8C). The abovementioned results indicated that NAR could promote the transcriptional regulation of ERα as a nuclear receptor and enhance the expression levels of downstream genes by directly binding to ERα.

Next, TAM, a selective ER modulator competitively binding to ERs, was employed to elucidate the role of ERα in NAR's effect on DFU. As detected by RT-qPCR, NAR enhanced the expression levels of Parkin and TFAM in HG-induced cells, while it was abolished by TAM (Fig. 8D). As displayed in Figs. 8E, S6H, and S6I, NAR diminished the levels of mitochondrial protein HSP60 and autophagy substrate P62. However, the abovementioned effects were blocked by TAM. NAR slightly increased the expression of VDAC1 and LC3II/LC3I ratio, which was further enhanced by combining TAM (Figs. 8E, S6J, and S6K). In parallel, TAM weakened the upregulation of NAR on the expression levels of anti-oxidative proteins, including NQO1 and SOD2 (Figs. 8E, S6L, and S6M). Immunofluorescence analysis revealed that the co-localization of LC3 and Tomm20 in HG-induced cells was preserved by NAR, which was inhibited upon TAM treatment (Fig. 8F). It was observed that NAR restored normal MMP and inhibited HG-induced overproduction of mitochondria-derived ROS, while had no effect in combination with TAM (Figs. 8G and H). The abovementioned results indicated that NAR promoted transcription of downstream estrogen response genes, including *Parkin*, through direct binding to ERα, thus exerting beneficial effects on cells under HG conditions.

## 4 Discussion

The present study revealed a novel mechanism by which NAR could accelerate diabetic wound healing. It was found that local application of NAR around the wounds of diabetic C57BL/6J mice accelerated wound healing by promoting epidermal proliferation, reducing skin aging, and suppressing oxidative stress, rather than in diabetic *Prkn*^*−*/−^ mice. Mechanistically, NAR promoted the translocation of Parkin to mitochondria by increasing both basal expression and autophosphorylation levels of PINK1. Moreover, NAR promoted Parkin-mediated mitophagy and upregulated the expression level of Parkin to maintain MQC and normal mitochondrial function, thus alleviating oxidative stress and promoting wound healing in DFU. The blocking of NAR binding to ERα or the deficiency of Parkin abolished the benefits of NAR.

This study demonstrated the crucial roles of Parkin and Parkin-mediated mitophagy in DFU. It was revealed that the healing effects of NAR on diabetic wounds are dependent on Parkin-mediated mitophagy. Several studies have demonstrated that Parkin and its mediated mitophagy contribute to the occurrence and progression of diabetic complications. The expression level of Parkin was downregulated in the dorsal root ganglion of diabetic rats, and the restoration of Parkin-mediated mitophagy could alleviate mitochondrial dysfunction and mitigate the symptoms of diabetic neuropathy [49]. Parkin expression level was significantly reduced in diabetic murine heart tissues, and Parkin knockout further aggravated mitochondrial and cardiac dysfunction in diabetic mice [50]. In addition, upregulating Parkin to promote mitophagy can improve myocardial ischemia-reperfusion injury in type 2 diabetes and reduce ROS accumulation [51]. However, the role of Parkin and its mediated mitophagy in the pathogenesis of DFU have yet to be reported. In the present study, a *Prkn*^*−*/−^ DFU mouse model was established, and it was revealed that the loss of Parkin could offset NAR's promoting effect on wound healing, suggesting that Parkin played a positive role in DFU wound healing. Parkin-deficient cells showed abnormal morphological characteristics with uneven cell size, shrinkage, and roundness. Upon induction with HG, Parkin-deficient cells were characterized by low confluency and further increased ROS accumulation, suggesting that Parkin exerts cytoprotective effects both under normal conditions and during stress. By upregulating Parkin expression level and facilitating mitochondrial localization of Parkin through NAR therapy, mitophagy was preserved, mitochondrial function was significantly restored (i.e., decreased MMP depolarization and decreased mtROS), and cellular oxidative stress was alleviated. Notably, siRNA-mediated Parkin depletion in HaCaT cells eliminated the abovementioned beneficial effects of NAR, suggesting that the protective effects of NAR on keratinocytes in the HG environment depend on Parkin. Our findings fill in the gap in the study of the relationship between Parkin and its mediated mitophagy and the pathogenesis of DFU, strongly suggesting that targeting Parkin-mediated mitophagy has excellent potential for DFU. In addition, NAR may be a therapeutic candidate for Parkin-deficient diseases, including DFU.

The present study also revealed the critical role of MQC in wound healing in DFU. Although MQC imbalance phenotypes, such as inhibition of mitochondrial biosynthesis, increased mitochondrial division, and impaired fusion, have been found in diabetic wounds [7,52], there is still no direct evidence that maintaining MQC balance benefits DFU wound healing. In this study, it was indicated that the expression levels of mitochondrial biosynthesis-related proteins, such as PGC-1α, NRF1, and TFAM, were downregulated in DFU, in the company of a reduction in mitochondrial protein levels and mitochondrial number. Moreover, HG induced a remarkable decline in MFN2 protein level, eventually leading to excessive mitochondrial fragmentation, represented by a decrease in the mean mitochondrial area. NAR therapy restored mitochondrial morphology and network complexity by rescuing levels of mitochondrial biosynthesis- and dynamics-related proteins. MQC balance re-maintained by NAR improved impaired mitochondrial function and keratinocyte function, ultimately promoting the wound healing of DFU. It was confirmed that maintaining MQC balance in keratinocytes is a valuable approach for treating DFU.

It was evidenced that ERα is the direct target of NAR, and by interacting with ERα, NAR has a strong promotion effect on the binding of ERα to the target promoter sequences, thus increasing the expression levels of downstream genes. The ERs antagonist ICI 182,780 (Fulvestrant) reverses NAR's anti-apoptotic and neuroprotective effects in β-amyloid protein-induced PC12 cells [53]. NAR promotes insulin secretion in primary isolated rat islets under HG conditions and increases glucose tolerance and plasma insulin levels in diabetic rats. These effects are blocked by a specific ERβ antagonist [54]. However, limited evidence supports the direct regulation of ERs by NAR. In this study, the CETSA results indicated that NAR significantly enhanced the thermal stability of ERα, rather than ERβ, indicating a direct interaction between NAR and ERα. To assess the transcriptional activity of nuclear ERα, luciferase reporter assay was performed to measure ERE activity. NAR significantly enhanced the activity of luciferase, whose effect was similar to E_2_. These results suggest that NAR promotes ERα nuclear translocation and ERE binding through direct interaction with ERα, thereby upregulating the transcription of target genes. The inconsistent results of NAR showing a preference for ERs' subtypes may be related to cell type and modeling differences. In conclusion, we provide direct, solid evidence that NAR could promote ERα nuclear translocation through its interaction with ERα, and thus exert its transcriptional regulatory function as a nuclear receptor.

These findings suggest that ERα plays a crucial role in NAR-mediated diabetic wound healing by regulating Parkin and maintaining MQC. Macrophage-specific ERα knockout reduced the anti-inflammatory effects of estrogen and delayed wound healing in ovariectomized mice [55]. Estrogen protects human umbilical cord blood mesenchymal stem cells (hUCB-MSCs) from oxidative stress and death induced by HG by promoting the nuclear transmutation of ERα, thereby improving the proliferation of hUCB-MSCs to promote wound healing in ovariectomized diabetic female mice [56]. However, there is limited evidence regarding specific downstream signaling pathways following ERα pharmacological activation in DFU. Loss of ERα reduces Parkin protein level in islet β cells and inhibits mitochondrial translocation of Parkin, resulting in the decreased mitophagy and mitochondrial dysfunction [57]. The correlation between ERα and Parkin in diabetes and its complications has never been reported. This prompted an investigation into the connection between ERα and Parkin, as well as their role in mitophagy during wound healing in DFU. A pharmacological approach was used, employing TAM as a selective ERs modulator. The results demonstrated that TAM reversed the effects of NAR on Parkin expression level and mitophagy. TAM treatment caused mitochondrial fragmentation and perinuclear distribution, which were associated with mitochondrial dysfunction. Additionally, TAM intensified cellular oxidative stress. These findings suggest that ERα plays a central role in NAR's promotion of Parkin-mediated mitophagy, and Parkin is an effective molecule through which ERα exerts a positive role in DFU wound healing. The results also suggest that NAR has crucial therapeutic value in metabolic diseases, such as DFU. The ERα signaling pathway is predominant in sexual dimorphism in metabolic diseases, especially in DFU.

However, there are limitations in the present study. Firstly, the binding domain of NAR with ERα has not been identified, warranting further investigation. Secondly, NAR's effects were tested in only one murine model. NAR is rapidly absorbed within 20 min of oral administration, with a median peak time of 3.5 h. Its median elimination half-life is 2.23 h, indicating that NAR exerts a rapid effect and is metabolized quickly in the human body [58]. A randomized, placebo-controlled, single-ascending-dose clinical trial showed no significant changes in some liver and kidney function markers in participants after a single ingestion of 900 mg NAR [59]. These pharmacokinetic characteristics and safety evaluations suggest that NAR possesses promising clinical potential. Therefore, conducting clinical trials using the local application of NAR to treat DFU is both necessary and valuable. Particular attention should be given to the gender and hormone levels of participants in clinical trials to further validate NAR's role and mechanisms.

## 5 Conclusions

The findings demonstrated that NAR accelerates wound healing of DFU. Mechanically, NAR activates Parkin-mediated mitophagy and maintains MQC balance via binding to ERα, thereby protecting mitochondrial function, thereby alleviating oxidative stress, inflammation, and senescence. The findings revealed that NAR may serve as a potential lead compound for anti-DFU drug development, and activating ERα to enhance Parkin-mediated mitophagy may be an effective strategy for treating DFU.

## CRediT authorship contribution statement

**Xin-Meng Zhou:** Writing – original draft, Methodology, Investigation, Formal analysis, Data curation. **Ying Yang:** Investigation, Data curation. **Dao-Jiang Yu:** Validation, Methodology, Formal analysis, Data curation. **Teng Xie:** Resources, Data curation. **Xi-Lu Sun:** Resources, Data curation. **Ying-Xuan Han:** Resources, Data curation. **Hai-Ying Tian:** Resources, Data curation. **Qing-Qing Liao:** Resources, Data curation. **Yu-Jie Zhao:** Resources, Data curation. **Yih-Cherng Liou:** Methodology. **Wei Huang:** Visualization, Methodology. **Yong Xu:** Methodology. **Xi Kuang:** Formal analysis, Visualization. **Xiao-Dong Sun:** Writing – review & editing, Supervision, Project administration, Funding acquisition, Formal analysis, Conceptualization. **Yuan-Yuan Zhang:** Writing – review & editing, Supervision, Project administration, Funding acquisition, Formal analysis, Conceptualization.

## Declaration of competing interest

The authors declare that there are no conflicts of interest.
