# Supplementary material for: Naringenin boosts Parkin-mediated mitophagy via estrogen receptor alpha to maintain mitochondrial quality control and heal diabetic foot ulcer
Source: J Pharm Anal. 2025 May 12;15(12):101333. doi: 10.1016/j.jpha.2025.101333 (PMC12756546; doi:10.1016/j.jpha.2025.101333)
Supplement: Multimedia component 1 [file mmc1.docx]

**Supplementary Table**

**Table S1** Primer information.

| Gene name | Forward primer (5'to3') | Reverse primer (5'to3') |
| --- | --- | --- |
| *PPARGC1A* | CAAGCCAAACCAACAACTTTATC | GCAAGAGGACTTCAGCTTTGG |
| *TFAM* | CCGAGGTGGTTTTCATCTGT | TCCGCCCTATAAGCATCTTG |
| *NRF1* | TACTCGTGTGGGACAGCAAG | AATTCCGTCGATGGTGAGAG |
| *PINK1* | CTGTCAGGAGATCCAGGCAATT | GCATGGTGGCTTCATACACAG |
| *PRKN* | GGAAGTCCAGCAGGTAGATCA | GTCCATCTTGCTGGGATGAT |
| *ACTB* | CATGTACGTTGCTATCCAGGC | CTCCTTAATGTCACGCACGAT |

Abbreviations: *PPARGC1A*, peroxisome proliferator-activated receptor gamma coactivator 1-alpha; *TFAM*, transcription factor A mitochondrial; *NRF1*, nuclear respiratory factor 1; *PINK1*, PTEN-induced putative kinase 1; *PRKN*, E3 ubiquitin-protein ligase parkin. *ACTB*, actin beta.

**Supplementary Figures and Figure captions**


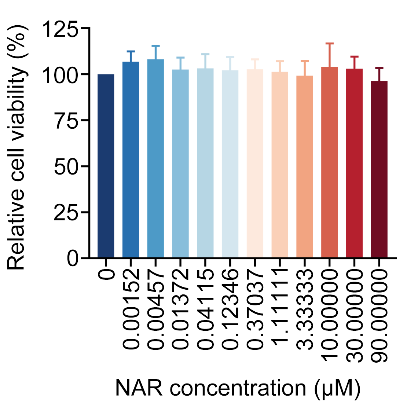


Fig. S1. Naringenin (NAR) has no cytotoxic effect on HaCaT cells. HaCaT cells were treated with various concentrations (μM) of NAR for 24 h, and then sulforhodamine B (SRB) assay was performed to examine cell viability. Data from three independent experiments are presented as the mean ± standard deviation (SD).


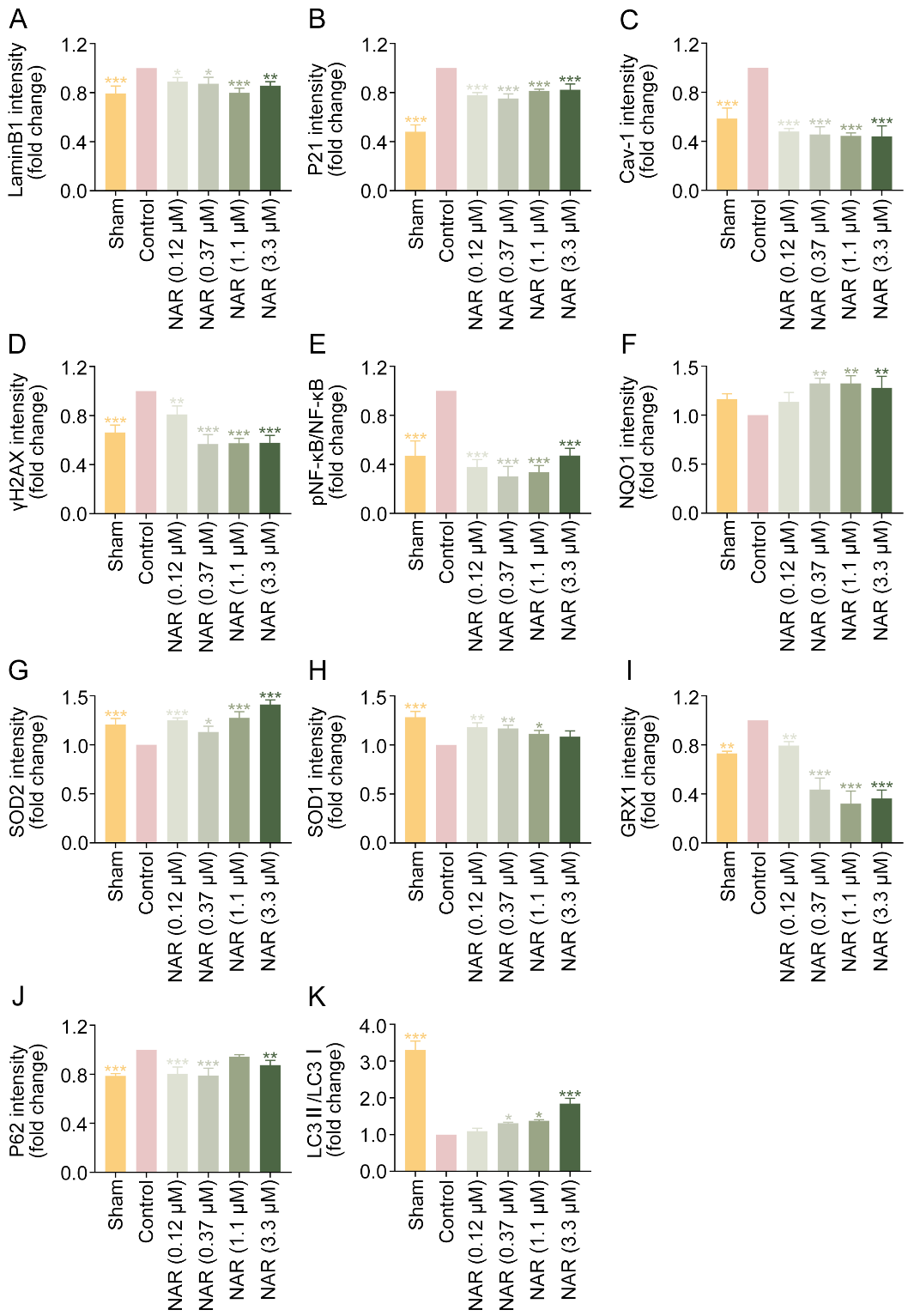


Fig. S2. Relative quantification of gray values of protein stripes in Figs. 2–3. (A–D) Quantitative results of the blots in Fig. 2D, LaminB1 (A), cyclin-dependent kinase inhibitor 1A (P21) (B), caveolin-1 (Cav-1) (C), and phospho-H2A histone family member X (γH2AX) (D). (E) Quantitative result of the blots of phospho-nuclear factor-κB (NF-κB) (S536)/NF-κB in Fig. 2E. (F–I) Quantitative results of the blots in Fig. 3D, NAD(P)H: quinone oxidoreductase 1 (NQO1) (F), superoxide dismutase 2 (SOD2) (G), superoxide dismutase 1 (SOD1) (H), glutaredoxin 1 (GRX1) (I). (J and K) Quantitative results of the blots in Fig. 3F, sequestosome-1 (P62) (J), microtubule-associated proteins 1A/1B light chain 3 (LC3) Ⅱ/LC3Ⅰ (K). Data from three experiments are presented as mean ± standard deviation (SD). The gray value of protein in the Control group was normalized to 1. ^*^*P* < 0.05, ^**^*P* < 0.01, ^***^*P* < 0.001 compared with the Control group, as examined by one-way analysis of variance. NAR, naringenin.


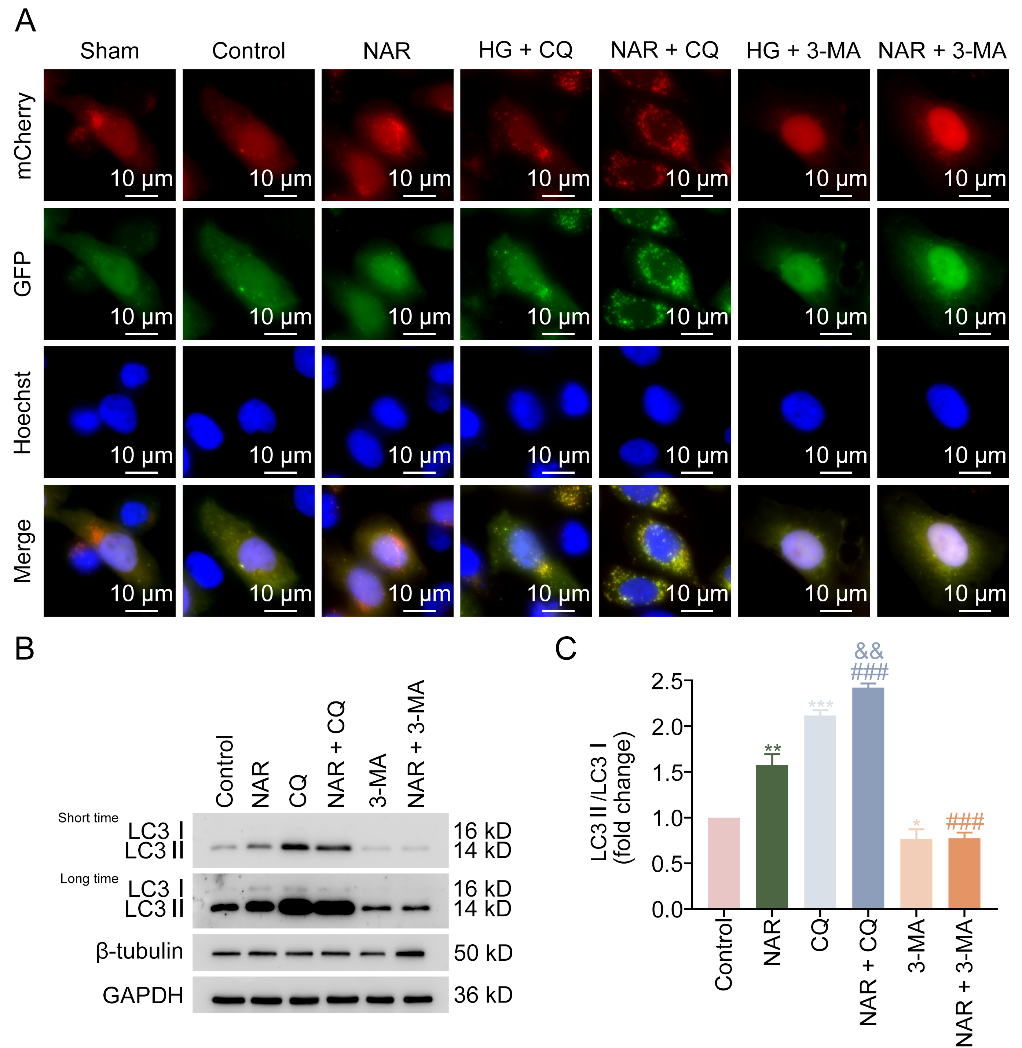


Fig. S3. Naringenin (NAR) restores the autophagic flux blocked by high glucose (HG). (A) HaCaT cells stably expressing mCherry-GFP-microtubule-associated proteins 1A/1B light chain 3 (LC3) were visualized by inverted fluorescence microscopy to evaluate the autophagic flux. The yellow dots shown by overlapping GFP and mCherry puncta represent autophagosomes. The red dots shown by the mCherry puncta represent autolysosomes. (B) HaCaT cells were induced with HG (50 mM) for 24 h, followed by incubation with NAR (3.3 μM) for 24 h in the presence or absence of chloroquine (CQ) (10 μM) or 3-methyladenine (3-MA) (10 mM). The expression level of LC3 was determined using Western blotting. (C) Quantitative analysis of the blots of LC3Ⅱ/LC3Ⅰ. Data from three experiments are presented as mean ± standard deviation (SD). The gray value of LC3Ⅱ/LC3Ⅰ in the Control group was normalized to 1. ^*^*P* < 0.05, ^**^*P* < 0.01, ^***^*P* < 0.001 compared with the Control group, as examined by Student’s t-test. ^###^*P* < 0.001 compared with the NAR group, as examined by Student’s t-test. ^&&^*P* < 0.01 compared with the CQ group, as examined by Student’s t-test. GAPDH, glyceraldehyde 3-phosphate dehydrogenase.


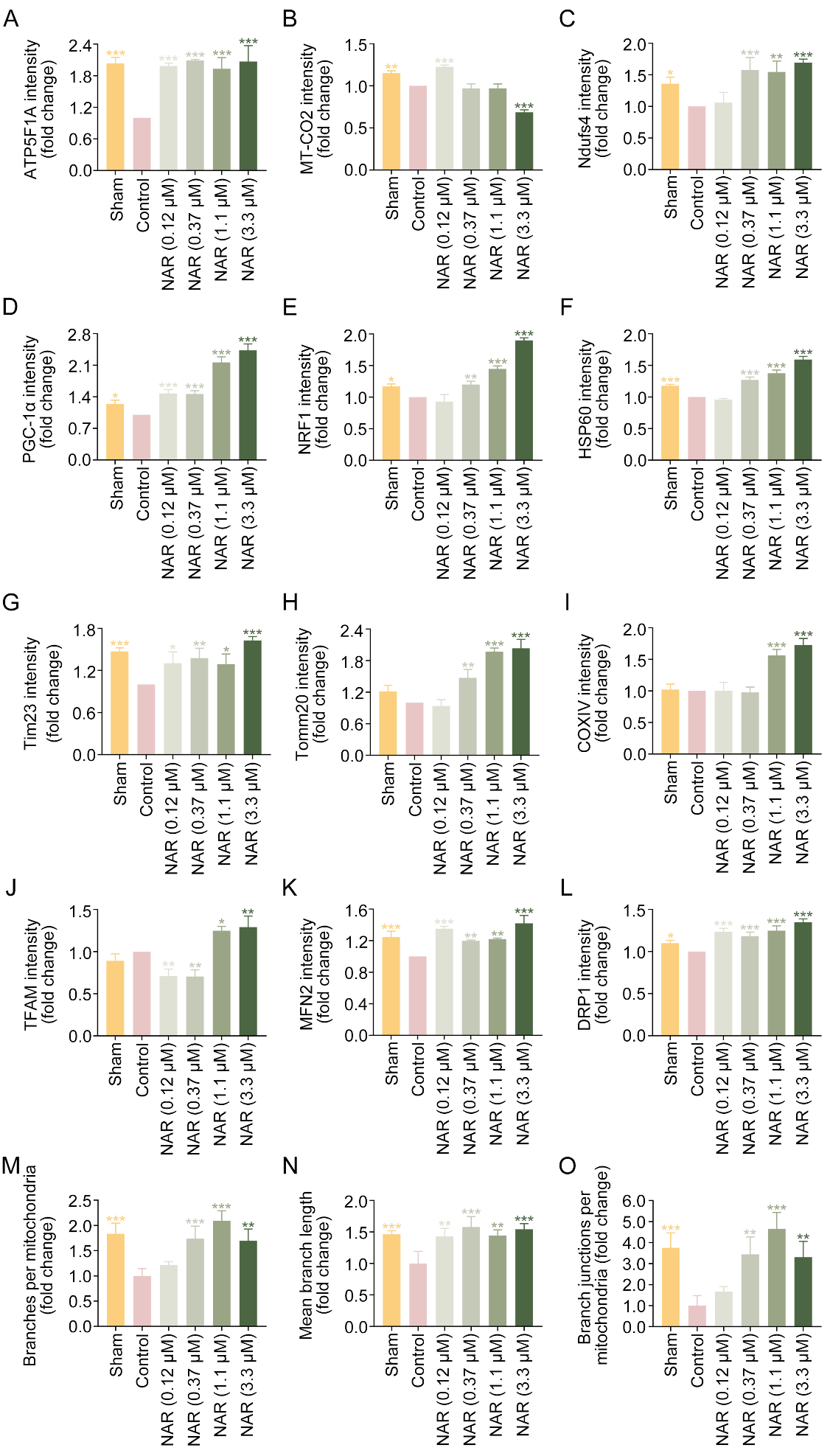


Fig. S4. Statistical analysis of protein stripes and fluorescence images in Figs. 4 (A–C) Quantitative results of the blots in Fig. 4A, ATP synthase F1 subunit alpha (ATP5F1A) (A), cytochrome c oxidase subunit 2 (MT-CO2) (B), NADH dehydrogenase (ubiquinone) iron-sulfur protein 4 (Ndufs4) (C). (D–J) Quantitative results of the blots in Fig. 4C, peroxisome proliferator-activated receptor gamma coactivator 1-alpha (PGC-1α) (D), nuclear respiratory factor 1 (NRF1) (E), heat shock protein 60 (HSP60) (F), translocase of inner mitochondrial membrane 23 (Tim23) (G),translocase of outer mitochondrial membrane 20 (Tomm20) (H), cytochrome c oxidase subunit 4 (COXⅣ) (I), and transcription factor A, mitochondrial (TFAM) (J). (K and L) Quantitative results of the blots in Fig. 4E, mitofusin 2 (MFN2) (K), dynamin-1-like protein (DRP1) (L). (M–O) Analysis of the branches per mitochondria (M), mean branch length (N), and branch junctions per mitochondria (O). Data are presented as mean ± standard deviation (SD). The value of the Control group was normalized to 1. ^*^*P* < 0.05, ^**^*P* < 0.01, ^***^*P* < 0.001 compared with the Control group, as examined by one-way analysis of variance. NAR, naringenin.


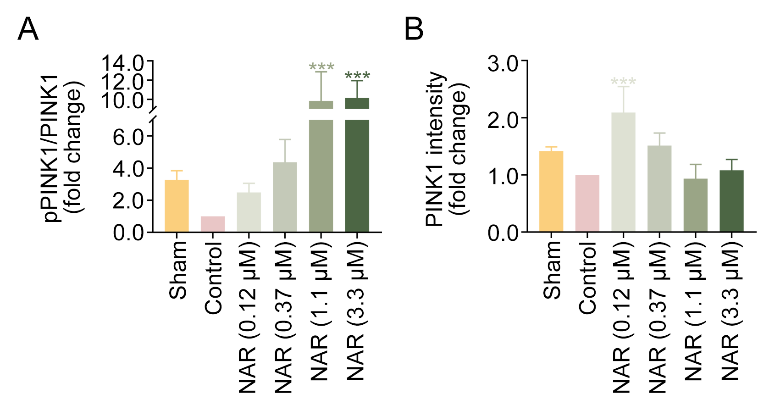


Fig. S5. Relative quantification of gray values of protein stripes in Fig. 6D, phospho-PTEN-induced putative kinase 1 (PINK1) (S228)/PINK1 (A), PINK1 (B). Data from three experiments are presented as mean ± standard deviation (SD). The gray value of protein in the Control group was normalized to 1. ^***^*P* < 0.001 compared with the Control group, as examined by one-way analysis of variance. NAR, naringenin.


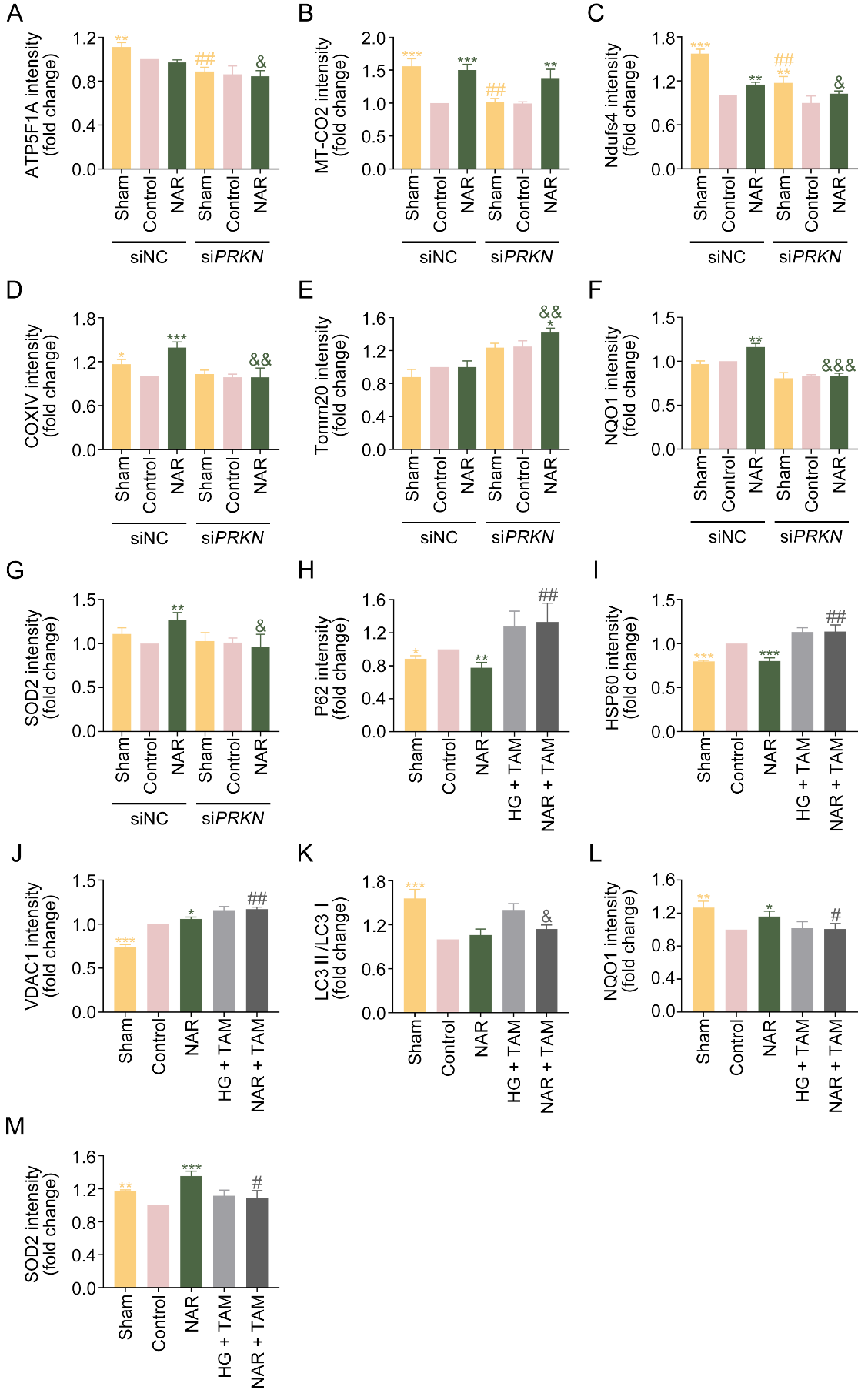


Fig. S6. Relative quantification of gray values of protein stripes in Figs. 6I and 8E. (A–G) Quantitative results of the blots in Fig. 6I, ATP synthase F1 subunit alpha (ATP5F1A) (A), cytochrome c oxidase subunit 2 (MT-CO2) (B), NADH dehydrogenase (ubiquinone) iron-sulfur protein 4 (Ndufs4) (C), cytochrome c oxidase subunit 4 (COXⅣ) (D), translocase of outer mitochondrial membrane 20 (Tomm20) (E), NAD(P)H: quinone oxidoreductase 1 (NQO1) (F), and superoxide dismutase 2 (SOD2) (G). Data from three independent experiments are presented as mean ± standard deviation (SD). The gray value of protein in the siNC-Control group was normalized to 1. ^*^*P* < 0.05, ^**^*P* < 0.01, ^***^*P* < 0.001 compared with the Control group in the same subject, as examined by one-way analysis of variance (ANOVA). ^##^*P* < 0.01 compared with the siNC-Sham group, as examined by Student’s t-test. ^&^*P* < 0.05, ^&&^*P* < 0.01, ^&&&^*P* < 0.001 compared with the siNC-naringenin (NAR) group, as examined by Student’s t-test. (H–M) Quantitative results of the blots in Fig. 8E, sequestosome-1 (P62) (H), heat shock protein 60 (HSP60) (I), voltage-dependent anion-selective channel protein 1 (VDAC1) (J), microtubule-associated proteins 1A/1B light chain 3 (LC3) Ⅱ/LC3Ⅰ (K), NQO1 (L), and SOD2 (M). Data from three independent experiments are presented as mean ± SD. The gray value of protein in the Control group was normalized to 1. ^*^*P* < 0.05, ^**^*P* < 0.01, ^***^*P* < 0.001 compared with the Control group, as examined by one-way ANOVA. ^#^*P* < 0.05, ^##^*P* < 0.01 compared with the NAR group, as examined by Student’s t-test. ^&^*P* < 0.05 compared with the high glucose (HG) + tamoxifen (TAM) group, as examined by Student’s t-test. siNC, negative control small interfering RNA; *PRKN*, E3 ubiquitin-protein ligase parkin.
